# Prediction and validation of novel SigB regulon members in *Bacillus subtilis* and regulon structure comparison to Bacillales members

**DOI:** 10.1186/s12866-022-02700-0

**Published:** 2023-01-18

**Authors:** Kah Yen Claire Yeak, Jos Boekhorst, Michiel Wels, Tjakko Abee, Marjon H J Wells-Bennik

**Affiliations:** 1 NIZO, Ede, The Netherlands; 2 Food Microbiology, Wageningen University and Research, Wageningen, The Netherlands; 3 Host Microbe Interactomics Group, Wageningen University and Research, Wageningen, The Netherlands; 4 Rijk Zwaan Breeding B.V, Fijnaart, The Netherlands

**Keywords:** SigB promoter binding motif, SigB consensus, General stress response, RsbRST, RsbQP, RsbKY, *Listeria monocytogenes*, *Staphylococcus aureus*

## Abstract

**Background:**

Sigma factor B (SigB) is the central regulator of the general stress response in *Bacillus subtilis* and regulates a group of genes in response to various stressors, known as the SigB regulon members. Genes that are directly regulated by SigB contain a promotor binding motif (PBM) with a previously identified consensus sequence.

**Results:**

In this study, refined SigB PBMs were derived and different spacer compositions and lengths (N_12_-N_17_) were taken into account. These were used to identify putative SigB-regulated genes in the *B. subtilis* genome, revealing 255 genes: 99 had been described in the literature and 156 genes were newly identified, increasing the number of SigB putative regulon members (with and without a SigB PBM) to > 500 in *B. subtilis.* The 255 genes were assigned to five categories (I-V) based on their similarity to the original SigB consensus sequences. The functionalities of selected representatives per category were assessed using promoter-reporter fusions in wt and Δ*sigB* mutants upon exposure to heat, ethanol, and salt stress. The activity of the P_*rsbV*_ (I) positive control was induced upon exposure to all three stressors. P_*ytoQ*_ (II) showed SigB-dependent activity only upon exposure to ethanol, whereas P_*pucI*_ (II) with a N_17_ spacer and P_*ylaL*_ (III) with a N_16_ spacer showed mild induction regardless of heat/ethanol/salt stress. P_*ywzA*_ (III) and P_*yaaI*_ (IV) displayed ethanol-specific SigB-dependent activities despite a lower-level conserved − 10 binding motif. P_*gtaB*_ (V) was SigB-induced under ethanol and salt stress while lacking a conserved − 10 binding region. The activities of P_*ygaO*_ and P_*ykaA*_ (III) did not show evident changes under the conditions tested despite having a SigB PBM that highly resembled the consensus. The identified extended SigB regulon candidates in *B. subtilis* are mainly involved in coping with stress but are also engaged in other cellular processes. Orthologs of SigB regulon candidates with SigB PBMs were identified in other Bacillales genomes, but not all showed a SigB PBM. Additionally, genes involved in the integration of stress signals to activate SigB were predicted in these genomes, indicating that SigB signaling and regulon genes are species-specific.

**Conclusion:**

The entire SigB regulatory network is sophisticated and not yet fully understood even for the well-characterized organism *B. subtilis* 168. Knowledge and information gained in this study can be used in further SigB studies to uncover a complete picture of the role of SigB in *B. subtilis* and other species*.*

**Supplementary Information:**

The online version contains supplementary material available at 10.1186/s12866-022-02700-0.

## Introduction

The general stress response (GSR) in bacteria constitutes a vital trait for cells to adapt to and survive conditions such as temperature change, nutrient depletion, or exposure to reactive oxygen species in natural niches.

The GSR in *Bacillus subtilis* and many Bacillales members is under the transcriptional control of the alternative sigma factor B (σ^B^ or SigB) [1]. Different environmental or nutritional signals, such as heat, ethanol, salt, and glucose starvation can induce the SigB-mediated GSR, resulting in the expression of SigB-dependent genes and the production of proteins to protect cells from injuries [2–4]. The production of these proteins provides general protection to the cells and confers resistance to multiple stresses, allowing for rapid adaption to changing environments, thereby enhancing the survival of vegetative cells in extreme habitats [5].

The genes/proteins with SigB-dependent expression are defined as the SigB regulon members. Those that are directly regulated by SigB contain a promoter binding motif (PBM) consensus sequence GTTTAA-N_15 (± 2 bp)_ – GGGTAT [6–8]. Those that are indirectly controlled by SigB do not have a SigB PBM and can either be controlled via SigB-dependent genes/proteins or regulated by other transcriptional regulators [8]. To date, 224 genes have been listed as members of the SigB regulon on Subtiwiki for *B. subtilis* [2, 3, 5, 9–13]*.* A recent study by Vohradsky et al. [8] further expanded the number to 411, with around 30% of the SigB regulon genes reported to lack a SigB PBM, and around 60–95% predicted to contain only a partial SigB PBM (i.e., only the − 35 or the − 10 promoter region, upstream of the AUG start codon).

Over time, more and more SigB regulon genes have been identified. This indicates that SigB regulatory networks are sophisticated and heavily interlinked with various other cellular mechanisms that are regulated by other transcriptional regulators. For example, SigB is indirectly involved in cellular responses such as sporulation and biofilm formation in *B. subtilis* [14, 15]. SigB-dependent genes may be expressed selectively to serve particular functions under certain conditions, *e.g.,* 36% of the 411 experimentally confirmed and predicted genes are expressed during spore germination and outgrowth in *B. subtilis* [8], and roughly half are needed to cope with physical stresses such as exposure to ethanol, butanol, salt, or high/low temperatures [5]. In *B. subtilis,* distinct sets of SigB-dependent genes are expressed under different environmental conditions in nature. This may hold true as well for other *Bacillus* species like *Bacillus cereus* and *Bacillus licheniformis,* or other Bacillales members that contain SigB. In members of the *B. cereus* sensu *lato* group – which contain a common set of genes - it was found that the presence of varying SigB promoters gave rise to a unique SigB regulon structure per species, influencing pathogenesis via different strategies [16]. It is anticipated that Bacillales members that inhabit different niches may have SigB regulon structures that are different from *B. subtilis* and that these may have evolved to be mediated by different SigB activation routes.

Currently, four SigB activation routes are known for Bacillales members, namely, I) the stressosome RsbRST (Rsb = Regulator of SigB); II) the bipartite RsbQP, III) the two-component RsbKY system, and IV) direct activation (here refer to as Rsb-independent), as reviewed in Pané-Farré et al. [17] and Rodriguez Ayala et al. [18]. The stressosome signaling complex is formed by RsbR and its paralogs (RsbRB, RsbRC, RsbRD, and YtvA), RsbS serine phosphatase, and RsbT serine kinase [17, 19, 20]. The stressosome becomes phosphorylated upon exposure to environmental stressors, and RsbT is then released to dephosphorylate the RsbU phosphatase, leading to further dephosphorylation of the RsbV anti-sigma factor antagonist [21]. The dephosphorylated RsbV uncouples the binding between the anti-sigma factor RsbW and SigB, promoting the transcriptional activation of SigB and subsequently the expression of SigB regulon genes [19, 22, 23]. Activation of SigB via RsbQP involves the signal transfer from the α-hydrolase activator RsbQ to the RsbP phosphatase under nutritional stress (*e.g.,* decrease in ATP, glucose starvation). RsbP then dephosphorylates RsbV, resulting in the same sequential SigB activation as for activation via the stressosome [24, 25]. The RsbKY two-component system includes the histidine sensor kinase RsbK and its cognate response regulator, RsbY [26]. By default, the methyltransferase (RsbM) methylates RsbK and negatively regulates SigB. Upon exposure to environmental/nutritional stressors, RsbK autophosphorylates and activates the RsbY phosphatase [27, 28], and subsequent SigB activation takes place in the same way as for the stressosome and the RsbQP module [26, 28, 29]. However, not all of the above-mentioned SigB activation systems are present in all Bacillales. Lastly, in low-temperature adaptation or nitrosative stress adaptation, SigB is activated independently from its regulators, RsbU, RsbP, and RsbV [30, 31].

To better understand the SigB regulon structures and functions in *B. subtilis*, this study employed *B. subtilis* 168 as a model, and used a newly derived SigB PBM to perform genome mining for novel SigB direct regulon members. The functionality of several predicted PBMs was verified using translational fusions to a reporter in a wild type (wt) and Δ*sigB* background. The SigB regulons in 18 different *B. subtilis* strains and 106 Bacillales genomes were also assessed and a Bacillales SigB PBM consensus sequence was obtained. Lastly, the absence and presence of the four SigB activation routes in the 106 Bacillales genomes were predicted.

## Materials and methods

### Sigma B (SigB) promoter binding motif (PBM) reconstruction

**Fig. 1:**
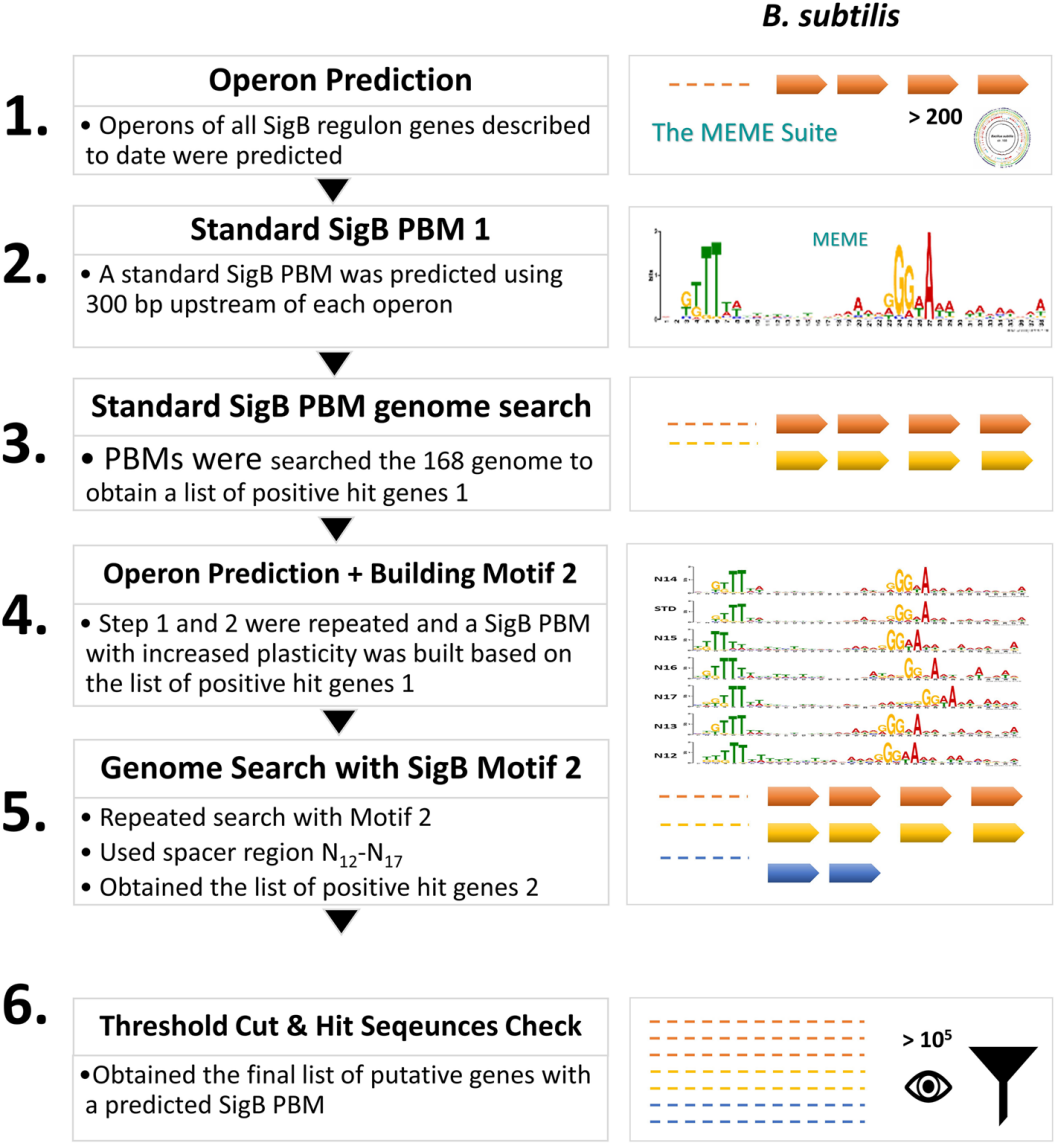
Flowchart of the reconstruction of SigB promoter binding motif for *Bacillus subtilis.* All SigB PBMs were predicted using the MEME Suite version 5.0.5 [35, 36], and MAST was used to screen the genome for a potential SigB PBM, as described by Wels et al. [32, 33]. The letter N indicates the number of base pairs present in the spacer region. STD stands for the standard motif, which was built based on all the listed SigB regulon genes on Subtiwiki up to October 2020 [13]. The *p*-value cut-off threshold, indicating the confidence level of the predicted motif, was set as 10^5^. All final sequences predicted for *B. subtilis* 168 were manually curated and listed in Supplementary Table S2

To identify novel SigB direct regulon members in *B. subtilis* 168, the SigB PBM was reconstructed as described by Wels et al. [32, 33] with slight modifications. In short, the respective operons of SigB regulon genes known to date in *B. subtilis* 168 were grouped according to their predicted operons (Supplementary Table S1). In total, 226 genes belonging to the SigB regulon were obtained for *B. subtilis* 168 on Subtiwiki [13], resulting in a compiled list with a total of 224 genes after removing duplicates. The operon structures of these genes were subsequently assessed and genes were allocated to the same operon when: 1) adjacent genes were on the same coding strand, 2) the intergenic region between adjacent genes was < 50 bp, and 3) no terminator was found between adjacent genes using Transterm (a tool to predict Rho-independent terminator) [34]. Regions 300 bp upstream of each operon and the full intergenic region (if < 300 bp) were then inspected to identify the SigB PBM, and used to derive a standard SigB PBM in MEME Suite [35, 36]. MEME was run using standard settings, with the following exceptions; −mod zoops (zero or one occurrence per sequence), −minw 10 (minimum width of 10), −maxw 50 (maximum width of 50), −dna (DNA molecule). The derived SigB PBM was used to repeat a search on the genome of *B. subtilis* 168 to obtain a new list of positive hit genes with a putative SigB PBM using the MAST search option in the MEME suite. The respective operons for these positive hit genes were predicted as aforementioned, and then a refined SigB PBM was built with MEME Suite. This refined SigB PBM with increased plasticity was used to search the genome of the 168 strain repeatedly, each with a different promoter spacer length, from N_12_ to N_17_. Promoter space length was increased/decreased by deleting or copying the least informative position in the position-specific scoring matrix (PSSM). The *p*-value indicating the confidence of predicted PBMs was set at 10^− 5^ (illustrated in Fig. 1). Each promoter hit sequence was manually curated (Supplementary Table S2). Different spacers were screened in this study as Vohradky et al. [8] indicated that genes controlled by SigB must contain both − 35 and − 10 binding motifs in the SigB PBM, and have a spacer length of 15 to 17 bp. Additionally, the similarity of the SigB PBM to the SigB consensus (indicated by the *p*-value) also took the nucleotide composition of spacers into account in this study. This is because the spacer compositions may influence the promoter binding strength and gene expression, and may promote co-recognition by different transcriptional regulators [37–39].

### Bacterial culturing conditions, media, chemicals, and DNA manipulations

All strains of *B. subtilis* and *Escherichia coli* used in this study were cultured in Lysogeny broth (LB) medium (Tritium Microbiologie, Eindhoven, The Netherlands), and propagated on LB agar plates unless stated otherwise. All incubations were performed at 37 °C, and all liquid cultures were incubated using shaking at 220 rpm. For standard DNA cloning, plasmids were prepared and isolated from TOP10 *E. coli* cells (Thermo Fischer Scientific, Bleiswijk, The Netherlands). Chemically competent *E. coli* cells were transformed via heat shock [40]. *B. subtilis* cells were transformed via natural competence in 1X MC competence medium (containing 200 μl of 10X MC plus 6.7 μl of 1 M MgSO_4_, 10 μl of 1% tryptophan, and 1.8 ml of sterile water). A stock solution of 10X MC was prepared with 14.036 g K_2_HPO_4_.3H_2_O, 5.239 g KH_2_PO_4_, 20 g glucose, 10 ml of 300 mM Na_3_C_6_H_5_O_7_, 1 ml of C_6_H_8_FeNO_7_, 1 g of casein hydrolysate, and 2 g of KC_5_H_8_NO_4_ to a total volume of 100 ml H_2_O. The amylase activity of *B. subtilis* transformants was tested on 1% starch plates and stained with iodine. For the β-galactosidase assay, *B. subtilis* was cultured in a C-minimal medium supplemented with 1 g/L glucose and 8 g/L potassium glutamate (CE) as described by Commichau et al. [41].

Oligonucleotides and KOD hot-start DNA polymerase were purchased from Merck (Zwijndrecht, The Netherlands). *B. subtilis* chromosomal DNA was isolated using the GenElute Bacterial Genomic DNA Kit (Merck). Plasmids were isolated using the GeneJET Plasmid Miniprep Kit (Thermo Fischer Scientific). PCR products were purified using the PCR Purification Kit (Qiagen, Hilden, Germany). All FastDigest restriction enzymes and T4 DNA ligase were purchased (Thermo Fischer Scientific) and used according to the manufacturer’s instructions. Bacterial culturing media were purchased (Tritium Microbiologie). DNA sequencing was performed by BaseClear B.V. (Leiden, The Netherlands).

### Plasmids and reporter strains construction

The plasmids and strains constructed and used in this study are listed in Supplementary Table S3. The *B. subtilis* Δ*sigB* mutant was constructed using the long flanking homology recombination method as described in Kunst and Rapoport [42] with the selective marker for chloramphenicol resistance amplified from pNZ5319 [43]. The *cre*-recombinase plasmid pDR244 (purchased from the *Bacillus* Genetic Stock Centre, Columbus, USA) was used to excise the chloramphenicol cassette at the *sigB* locus [44] to obtain a Δ*sigB* clean deletion mutant (BY47).

Plasmids pCY22, pCY23, pCY26, pCY27, pCY31, pCY32, and pCY33 were constructed, containing translational reporter fusions of P_*yaaI*_, P_*ywzA*_*,* P_*pucI*_, P_*gtaB*_, P_*ylaL*_*,* P_*ykaA*_*,* and P_*ygaO*_, respectively (~ 150 bp upstream fragment), to *lacZ*. These SigB PBMs were selected as representative sequences as predicted in section “*Sigma B (SigB) promoter binding motif (PBM) reconstruction*” and were grouped into five categories irrespective of the spacer lengths: Category I (P_*rsbV*_): exact match at − 35 and − 10 regions; Category II (P_*ytoQ*_, P_*pucI*_): exact match either at − 35 or − 10 region; Category III (P_*ylaL*_, P_*ygaO*_, P_*ykaA*_): conserved GTTT at − 35 and NGG at − 10 region; Category IV (P_*yaaI*_): less conserved motif with high *p*-values; and Category V (P_*gtaB*_): with a duplicated − 35 or − 10 region. *RsbV* is a well-known SigB-induced gene and P_*rsbV*_ was selected as a positive control because the PBM has a perfect match to the reported SigB consensus. P_*ytoQ*_ and P_*pucI*_ were selected because both have conserved sequences at the − 35 and − 10 regions, but P_*pucI*_ has a longer spacer of N17, and P_*ytoQ*_ has the same spacer length as the consensus, which is N14. These two PBMs were selected to check whether the difference in spacer length affects the promoter binding activity. For category three: P_*ylaL*_, P_*ygaO,*_ and P_*ykaA*_ were selected because of the different spacer lengths of N16, N14, and N12, respectively. P_*ywzA*_ was selected because the gene *ywzA* has previously been reported by Petersohn et al. [3] to contain a SigB PBM, but the predicted PBM has not been tested so far. P_*yaaI*_ (category IV) was selected because of a deviated third nucleotide position in the − 35 region from guanine to cytosine and has a higher *p*-value than the predicted PBMs in category III. P_*gtaB*_ was selected because of the duplicated GTTTAA regions, and the predicted promoter sequence differed from the previous reports [2, 3].

The predicted SigB PBM (~ 150 bp in length) upstream of each mentioned gene, including the native ribosomal binding site, was amplified by Polymerase Chain Reaction (PCR) using the corresponding oligonucleotides as listed in Supplementary Table S4. Each amplified fragment was ligated into either pDG1728 (purchased from BGSC) or pAC7 *amyE* integration plasmid (courtesy of Dr. J. Stülke, University of Goettingen, Germany) with the restriction enzymes *EcoRI* and *BamHI* [45]. Additionally, the plasmid pBP638 (courtesy of Dr. F. Commichau, Cottbus-Senftenberg University, Germany) was used to study the activity of P_*ytoQ*_ and the plasmid pNW2205 (P_*rsbV *_*-lacZ*) (courtesy of Dr. N. Stanley Wall, University of Dundee, UK) was used as a positive control in the β-galactosidase-assay.

The constructed plasmids were transferred into chemically competent TOP10 *E. coli* cells using heat shock, and transformants were selected on LB agar supplemented with 100 μg/ml carbenicillin. Plasmids were isolated from the positive *E. coli* colonies, sequenced, and subsequently introduced into *B. subtilis* 168 wt and Δ*sigB* mutant (BY47) cells via natural competence. *B. subtilis* transformants were selected on Brain Heart Infusion (BHI) agar supplemented with antibiotics (either 250 μg/ml spectinomycin or 10 μg/ml kanamycin). Activities of the promoters in response to stresses known to trigger SigB activation were investigated.

### β-galactosidase reporter assay

The activity of a promoter in vivo was determined using the β-galactosidase assay. *B. subtilis* strains carrying promoter-*lacZ* reporter fusions were grown in C-Glc medium [41], supplemented with 250 μg/ml spectinomycin or 10 μg/ml kanamycin. Overnight cultures in C-Glc were used to inoculate fresh C-Glc medium to an optical density of 0.05 at 600 nm (OD_600_), and allowed to grow to OD_600_ ~ 0.35. Cells were then divided into different portions and subjected to either ethanol stress (4%), NaCl (6%), or heat (upshift from 37 °C to 48 °C) for 10 min. The time point of 10 min was selected because the β-galactosidase activities did not change at different time points, *i.e.,* 5 min, 10 min, 15 min, and 20 min after exposure to stressors using the P_*rsbV*_*-lacZ* positive control and the *P*_*gtaB*_* -lacZ* (data not shown). Moreover, the time point of 10 min was selected to prevent the influence of increased promoter activities when cells in the culture approach or enter the stationary phase. Even if growth is limited, *e.g.,* in 4% ethanol, cells may grow slowly, so a later time point at 30 min or 60 min would not be ideal. The experimental setting has a limitation to validate promoters that require a longer time to respond. Cell pellets were collected before and after stress treatment and stored at − 20 °C. Quantitative studies of *lacZ* expression in *B. subtilis* were performed as described previously by Stannek et al. [46]. Briefly, cells were thawed and lysed with 400 μl Z-buffer (0.48 mM Na_2_HPO_4_. 2H_2_O; 0.32 mM NaH_2_PO_4_; 0.08 mM KCl; 8 μM MgSO_4_; 0.4 mM ß-mercaptoethanol; 200 μg lysozyme and 200 μg DNAse I) for 1 hour (h) at 37 °C. Lyzed cultures were centrifuged at 17,000 g to remove cell debris, and 100 μl of cell-free crude extract per sample was transferred into a new Eppendorf tube. 700 μl of Z-buffer without ß-mercaptoethanol was mixed with the 100 μl of crude extracts and incubated for 5 min at 28 °C. 800 μl of Z-buffer without ß-mercaptoethanol was used as a reference. Subsequently, 200 μl of 4 mg/ml of ortho-nitrophenyl-β-galactoside (ONPG) was added to all the crude extracts and the reference and allowed to react at 28 °C. When the cell extract turned visibly yellow, 500 μl of 62.5 mM Na_2_CO_3_ was added to stop the reaction. The absorption of samples at λ = 420 nm (absorption of O-nitrophenol) was measured. The protein concentration was determined via the Bradford assay [47, 48] at λ = 595 nm using the commercial Roti®-Quant Bradford solution (Carl Roth, Karlsruhe, Germany) according to the manufacturer’s protocol. The absorbance at A_595nm_ and A_420nm_ was corrected with the blank without cells. The specific ß- galactosidase activity (indicating the SigB activity) in Miller Units (MU)/mg protein was calculated using the formula:

$$\frac{\textrm{Units}}{\textrm{mg}\ \textrm{protein}}=1000\ \textrm{x}\frac{\ \textrm{A}420\ \textrm{nm}-\left(1.75\ \textrm{x}\ \textrm{A}550\textrm{nm}\right)}{\Delta \textrm{T}\ \textrm{x}\ \textrm{V}\ \textrm{x}\ \textrm{A}595}$$

One unit of β – galactosidase = the amount of enzyme produced to hydrolyze the chromogenic substrates ONPG to one nmol of O-nitrophenol (absorbs light at λ = 420 nm) per minute at 28 °C. V = 0.1 ml. Absorbance at 550 nm represents the scatter from cell debris, and multiplication with 1.75 gives the approximation of the scatter observed at 420 nm.

### Bacillales core genome phylogenetic tree reconstruction, species-specific SigB PBM, and regulon structure prediction

To better understand the SigB regulon structure and function in *B. subtilis* and Bacillales members, a core genome phylogenetic tree of 18 *B. subtilis* strains (other than strain 168) and 106 Bacillales genomes was reconstructed (Supplementary Table S5). Species-specific SigB PBMs and SigB regulons were predicted and a Bacillales SigB consensus was derived as described below.

#### *B. subtilis* wild isolate strains and Bacillales members selection

The Bacillales members were selected when SigB operon genes had been described [17] and when they had been characterized for other properties (not necessarily related to SigB), such as producing high heat-resistance spores [49] or acid-tolerance [50]. The genomes of strains analyzed in this study included strains of *B. subtilis* of different origins (18 plus reference strain 168), *Bacillus amyloliquefaciens* (4)*, Bacillus vallismortis* (4)*, Bacillus licheniformis* (11), *Bacillus velenzensis* (1), *Bacillus cereus* (33), *Bacillus coagulans* (5)*, Bacillus thermoamylovorans* (5), *Bacillus pumilus* (6)*, Anoxybacillus* (6), *Bacillus sporothermodurans* (1)*, Geobacillus* (14), *Parageobacillus* (7), *Caldibacillus* (2)*, Paenibacillus* (2), *Listeria* (3) and *Staphylococcus* (3) which were extracted from the NCBI bacterial genome database, available at (https://www.ncbi.nlm.nih.gov/genome/microbes/).

#### Core phylogenetic tree reconstruction and absence/presence of *B. subtilis* SigB regulon members in other Bacillales members

Orthologous groups were constructed using OrthAgogue (PMID24115168) [51]. Orthologous protein sequences with exactly one copy in all 125 Bacillales genomes were aligned with Muscle (Edgar, R.C. Nucleic Acids Res 32(5), 1792–97) and a core genome phylogenetic tree was constructed from the concatenated alignments via PhyML [52, 53]. Subsequently, all genes belonging to the SigB regulon in *B. subtilis* 168 [13] were selected as genes of interest (GOI) (Supplementary Table S1). The absence or presence of these GOI in the other 124 genome sequences was predicted via genome mining (Supplementary Table S5). Locus tags or gene symbols were used to identify the corresponding orthologous group of each GOI. Then, all GOI were clustered using the 1-(Spearman rank correlation of the gene copy number in each genome) as a distance measure, and neighbor-joining as the clustering algorithm (Phylip package, (http://evolution.genetics.washington.edu/phylip.html). Lastly, the absence or presence of gene functions in a genome was predicted based on the absence or presence of one or more orthologs in that genome. The phylogenetic tree (“MLST trees”) heat map was visualized using iTOL (PMID27095192) [54].

#### Species-specific SigB PBMs and SigB regulon structures prediction for other Bacillales genomes

**Fig. 2:**
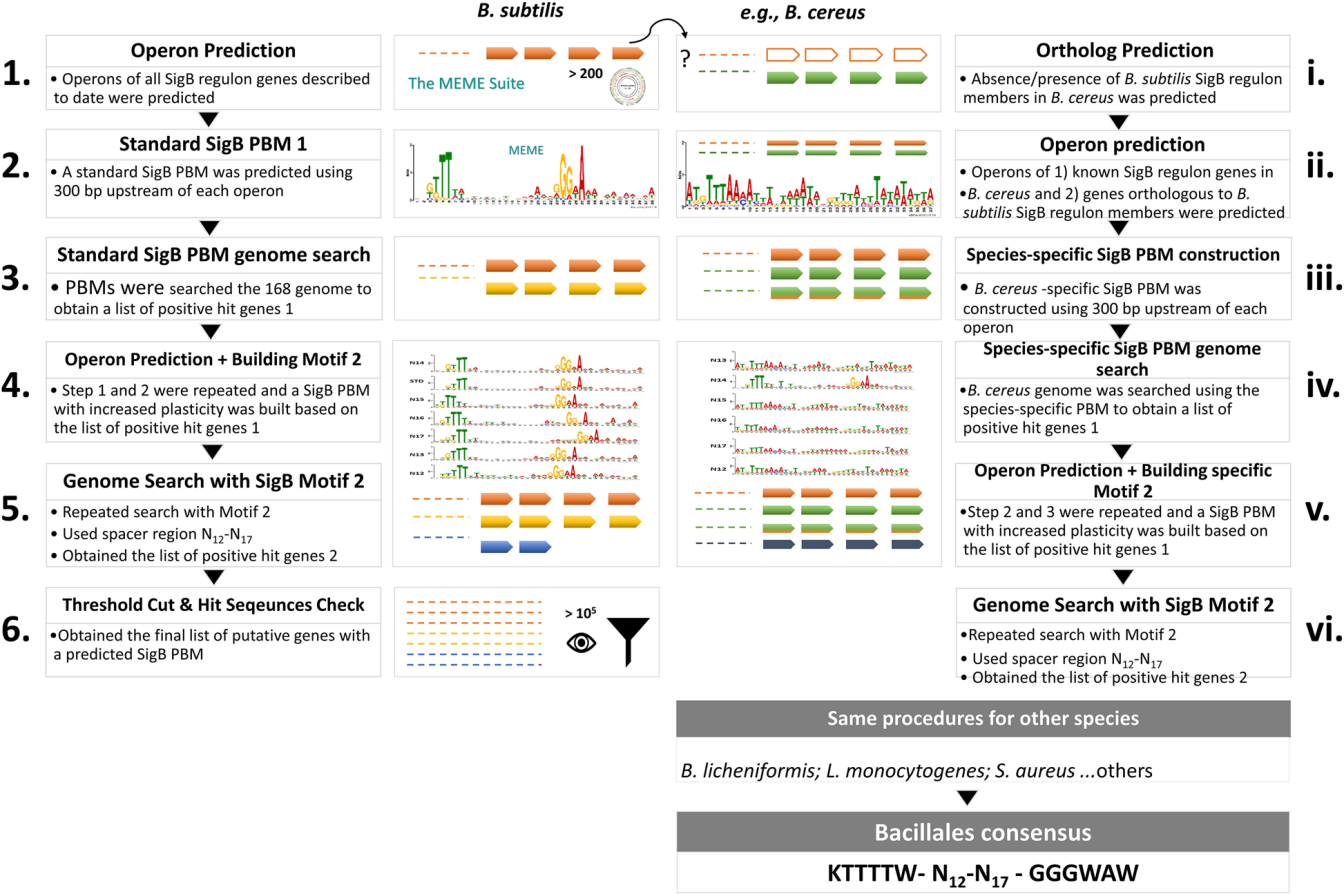
Flowchart of the reconstruction of species-specific SigB promoter binding motif for other species belonging to the order of Bacillales. All SigB PBMs were predicted using the MEME Suite version 5.0.5 [35, 36], and MAST was used to screen the genome for a potential SigB PBM, as described by Wels et al. [32, 33]. The letter N indicates the number of base pairs present in the spacer region. STD stands for the standard motif, which was built based on all the listed SigB regulon genes on Subtiwiki up to October 2020 [13]. The Bacillales SigB promoter binding motif consensus was acquired from the predicted species-specific SigB PBM from the 125 Bacillales genomes, including the *B. subtilis* 168 strain

The predicted orthologs of the *B. subtilis* 168 SigB regulon members in 18 other *B. subtilis* strains and 106 Bacillales genomes (identified as described in section “*Core phylogenetic tree reconstruction and absence/presence of B. subtilis SigB regulon members in other Bacillales members*”) were established and used to derive a species-specific SigB PBM via procedures reported in section “*Sigma B (SigB) promoter binding motif (PBM) reconstruction*” (for an example illustrated for constructing the *B. cereus* SigB PBM see Fig. 2, step ii and iii). Subsequently, the species-specific SigB PBM was employed to screen for genes with potential SigB PBMs in the respective species. Operons for the new positive hit genes were predicted, and the promoter regions of these genes were used to build the species-specific SigB PBM 2 (step v, Fig. 2). Then, the genome of each species was screened repeatedly with the improved species-specific SigB PBMs with different promoter spacer lengths (N_12_ to N_17_) as described in section “*Sigma B (SigB) promoter binding motif (PBM) reconstruction*”, respectively. The screening thus resulted in a list of genes with putative SigB PBMs per species, forming the predicted SigB regulon for each inspected Bacillales genome. The overall presence/absence of the predicted species-specific SigB regulon members was cross-checked between all 125 genomes that were analyzed (Supplementary Table S6). The genome tree heat map for 125 Bacillales members (including *B. subtilis* 168) was generated using GENESIS 1.7.7 [55] (Supplementary Fig. S1).

### Bacillales Sigma B (SigB) sensing modules prediction

To evaluate the ability of various Bacillales to employ different SigB signaling modules, the occurrence of the three known SigB sensing modules (RsbRST, RsbQP, and RsbKY) was evaluated in 125 Bacillales genomes as described in section “*Core phylogenetic tree reconstruction and absence/presence of B. subtilis SigB regulon members in other Bacillales members*”. Briefly, genes encoding the proteins involved in these three known SigB signal transduction pathways for Bacillales [2–5, 9, 10, 12, 16, 17, 26, 56, 57], and the SigB regulators *rsbX* and *rsbM* were set as the GOI. The absence/presence of these GOI in 125 genomes was predicted via genome mining to check for the absence or presence of an orthologous protein (“*Core phylogenetic tree reconstruction and absence/presence of B. subtilis SigB regulon members in other Bacillales members*” section (Supplementary Table S7) and a heat map was generated as mentioned in section “*Core phylogenetic tree reconstruction and absence/presence of B. subtilis SigB regulon members in other Bacillales members*” and visualized with iTOL.

## Results and discussion

### Two-step SigB promoter binding motif derivation in *B. subtilis* 168

**Fig. 3:**
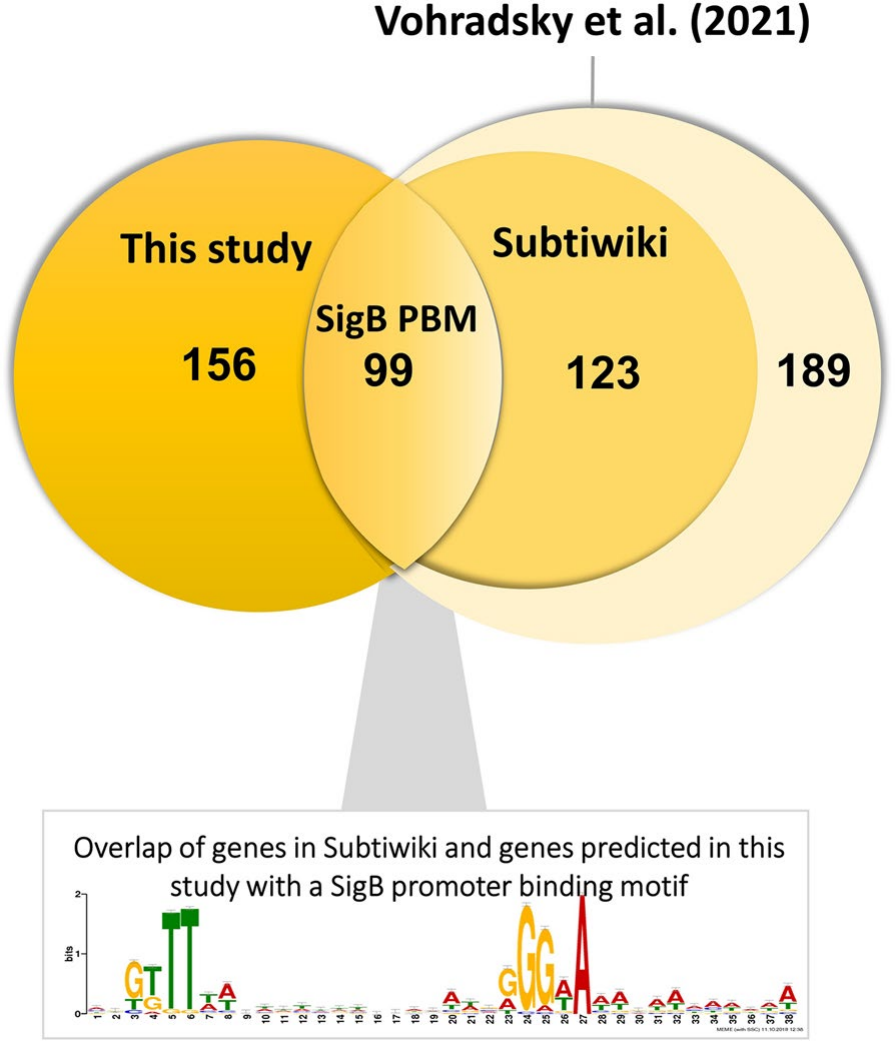
Novel SigB regulon genes detected for *Bacillus subtilis.* 255 SigB regulon genes with SigB PBMs were predicted, 99 overlapped with regulon genes already reported in literature, and 156 were newly identified in this study

Based on the analysis of the *B. subtilis* genome 168 using the refined SigB PBM (see section “*Sigma B (SigB) promoter binding motif (PBM) reconstruction*”), 255 genes (some belonging to the same operon) with a putative SigB PBM were predicted, indicating that they may be directly regulated by SigB (Supplementary Table S2). Of these, 99 overlap with SigB regulon members described in the literature: 74 were listed as SigB regulon members on Subtiwiki [13] (Fig. 3), and an additional 25 were recently reported by Vohradsky et al. [8]. Thus, a total of 156 of the 255 genes with a predicted SigB PBM are novel putative SigB regulated genes that were not identified in earlier studies [2–5, 8–12]. To avoid possible false positives in this prediction, each of the predicted sequences was manually checked (Supplementary Table S2).

The 255 genes that were predicted to have a SigB PBM in this study were grouped into five categories (Supplementary Table S2). Category I (green) contains 6 out of the 255 predicted genes with SigB PBMs that have an exact matching sequence to the previously reported SigB consensus motif (GTTTAA- N_15 (± 2 bp)_ -GGGTAT) [6–8], demonstrating that a very small number of the predicted genes have Category I SigB PBM. These are the well-known SigB-regulated genes, *i.e., sigB* itself, the anti-SigB antagonist *rsbV,* the serine protein kinase *rsbW,* the phosphoserine phosphatase *rsbX,* the general stress gene *ctc,* and the acetyltransferase *yjbC.* These genes with the category I SigB PBM and spacer length of N_14_ (except *yjbC* with N_13_) have been shown to have extensive differential regulation under various conditions that lead to the induction of SigB [2–4, 9].

Category II (orange) contains 27 out of 255 genes with a − 35 binding motif that is identical to the consensus motif GTTTAA and only 1 to 2 base pairs variations in the − 10 binding motif, or a − 10 binding motif that is identical to the consensus motif GGGTAT and only 1 to 2 base pairs variations in the − 35 binding motif. This category of SigB PBMs deviates the least from the SigB consensus and is likely easily recognized by SigB. Many general stress genes such as the glucose starvation gene *gsiB* have the category II SigB PBMs (Supplementary Table S2). Eleven newly identified genes in this category were not listed in Subtiwiki. Of these, *yjlB* was also recently discovered as SigB dependent by Vohradsky et al. [8], who identified SigB PBMs with − 35 and − 10 binding sites with spacers of 13–15 bp (which they referred to as so-called Class I promoters). The genes *pgcA, phoH,* and *yqhY* that were reported by Vohradsky et al. [8] were not identified in our study, likely due to the use of different settings for the promoter searches. Moreover, the length of the spacer was up to 50 bp in the study of Vohradsky et al. [8].

Most of the genes (137 out of 255) detected in this study contain Category III (yellow) SigB PBMs, which have the conserved GTTT bases at the − 35 binding site and GG at the − 10 binding site in the promoter region. Many of these SigB regulon genes (~ 50%) were identified in earlier studies [2–5, 9], listed as SigB regulon genes in Subtiwiki, or reported by Vohradsky et al. [8]. These findings imply that SigB can recognize the binding motif well as long as the bases at the − 35 and − 10 binding sites are conserved, and the length of the spacer is between N_12_ and N_17._

Category IV (grey) contains 76 genes with low level conserved SigB PBMs which have *p*-values close to the cut-off threshold (“*Sigma B (SigB) promoter binding motif (PBM) reconstruction*” section), likely indicating the presence of binding sequences for SigB and other transcriptional regulators. Several genes that are known to be coregulated by SigB and at least one other regulator were found in this group. These include, for instance, *secG* (preprotein translocase subunit), *yaaI* (general stress protein), and *plsC* (acylglycerolphosphate acyltransferase, biosynthesis of phospholipids). The *plsC* gene is known to be regulated by both SigB and FapR (a fatty acid synthetic gene repressor) to maintain membrane homeostasis [58]. Moreover, part of the SigB regulon overlaps with other regulons; it has been reported that SigB regulon members can be controlled by SigB and other regulators of which 36 have been reported (SigB and/or different regulators at different cellular states) [8, 13]. Therefore, the existence of genes that have less conserved SigB PBM or that do not have a SigB PBM but show SigB dependency suggested that cells may fine-tune response to integrate multiple signals in a variety of conditions.

Category V (blue) shows 9 out of 255 genes containing SigB PBM that deviated the most from the SigB consensus motif but were shortlisted likely due to the presence of either a duplicated − 35 or − 10 binding site or an occurrence of a single perfect − 35 or − 10 binding site (Supplementary Table S2). A previously described SigB regulon member, *gtaB* [3, 9], encoding the UTP-glucose-1-phosphate uridylyltransferase (a general stress protein), was found in this category. The duplicated GTTT region in the SigB PBM of *gtaB* might be the reason why the SigB PBM of *gtaB* had a low *p*-value (indicating higher similarity to the SigB consensus), suggesting that *gtaB* may be regulated by multiple regulators, via a versatile promoter sequence. To confirm this hypothesis, the predicted *gtaB* SigB PBM sequence was submitted to the database of transcriptional regulation in *B. subtilis* (DBTBS), and binding sequences of two other regulators SigA and DegU were found (Table 1). In the study of Vohradsky et al. [8], this gene was described to have a Class I promoter (i.e contained both − 35 and − 10 binding sites) and the SigB PBM was indicated at 96 bp upstream of ATG. However, a manual check on the genome of the strain 168 did not find a − 35 binding site.

**Table 1:**
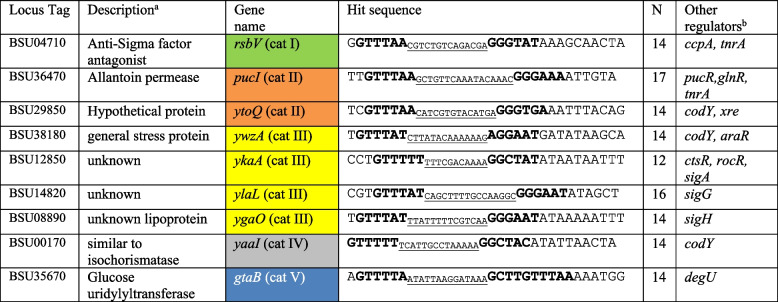
Nine selected promoters with predicted SigB promoter binding motifs.

*tnrA* Global nitrogen regulator. Negatively regulates glnR

*ccpA* Catabolite repressor or a positive regulator of genes involved in excretion of excess carbon

*glnR* Repressor of glutamine synthesis

*xre* Repressor of a phage-like bacteriocin, PBSX (phibacin damaged-prophage)

*araR* Repressor of the L-arabinose metabolic operon

*rocR* Activator of arginine utilization operons

*ctsR* Repressor of class III heat shock genes

*sigG* Transcription of sporulation genes

*sigH* Transcription of early stationary phase genes (sporulation/competence). Not active in lab strains due to a mutation (V117A)

*degU* Two-component response regulator, regulation of degradative enzyme and other adaptive responses

*codY* Repressor in response to branched-chain amino acid limitation

^a^ description based on Subtiwiki [13]

^b^predicted regulators on the DBTBS database [6]

### Experimental validation of predicted SigB promoter binding motifs

To further validate these in silico results, promoters of genes belonging to the five different assigned categories were selected and their SigB-dependent activities were studied using a promoter-reporter approach in a wild type (wt) and *ΔsigB* background.

**Fig. 4:**
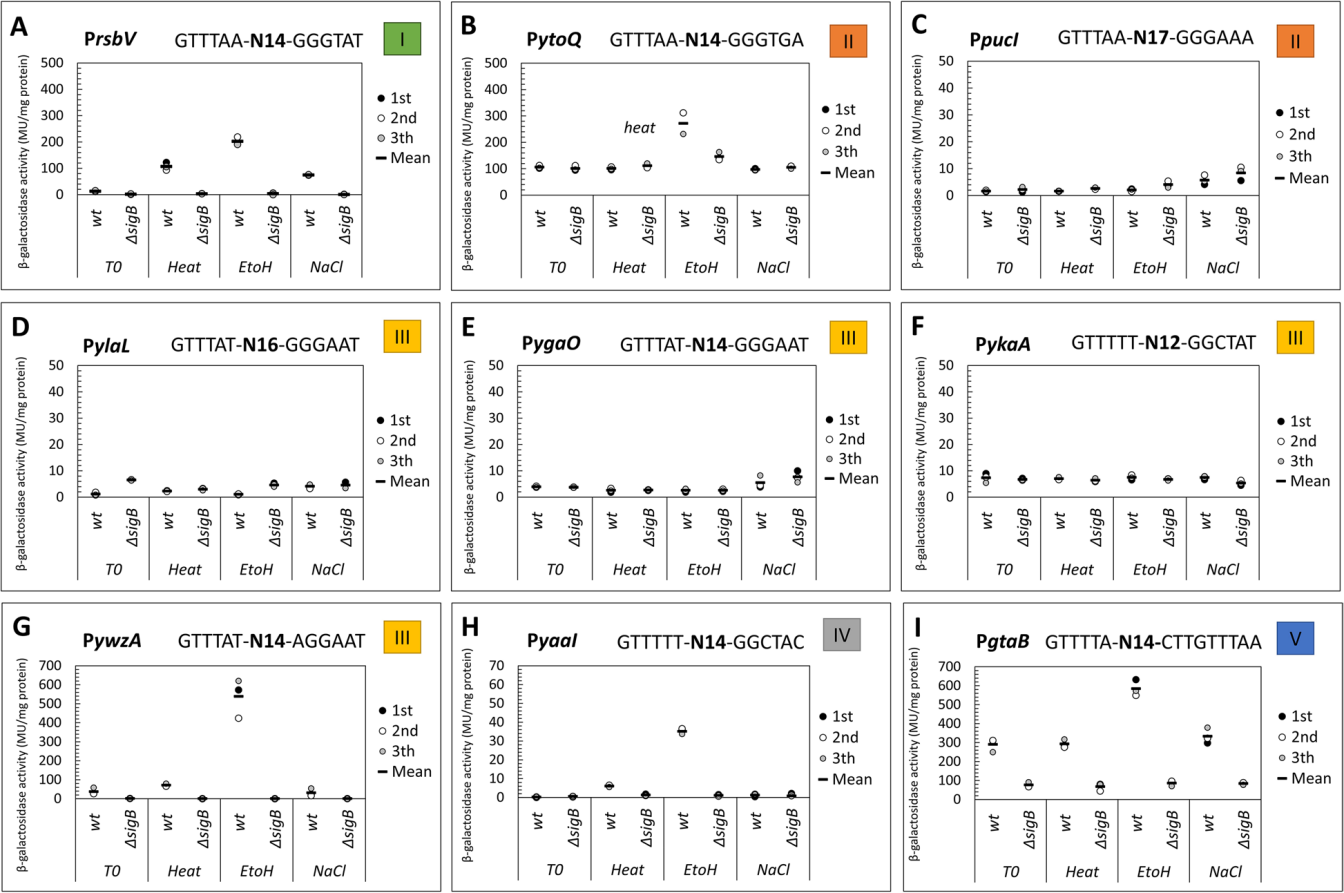
β-galactosidase activities of Category I-V predicted SigB promoters that were translationally fused to *lacZ* upon exposure to heat, ethanol, and salt stress. β-galactosidase activities of Category I-V predicted SigB promoters that were translationally fused to *lacZ* were investigated upon exposure to heat (37 °C > 48 °C), 4% (v/v) ethanol, and 6% (v/v) NaCl in wt cells and in the Δ*sigB* mutant of *B. subtilis*. Each data point represents a biologically independent replicate, and the bar indicates the average value for the three independent experiments. β-Galactosidase (LacZ) activities are presented in Miller units per milligram protein. The color-coded box at the right of each graph indicates the different categories to which the promoters belonged, based on the confidence level of the predicted PBM. Category I (green): the predicted PBM has the exact match at both − 35 and − 10 regions. Category II (orange): the predicted PBM has either an exact match at the − 35 region, with 1–2 bp variations at − 10, or vice versa. Category III (yellow): the predicted PBM has conserved GTTT bases at the − 35 region and the GG bases at the − 10 region. Category IV (grey): low-level homology compared with the conserved motif with borderline *p*-values; and Category V (Blue): with a duplicated − 35 or − 10 region. **A** P_*rsbV*_-*lacZ* activities; **B** P_*ytoQ*_ -*lacZ* activities; **C** P_*pucI*_ -*lacZ* activities; **D** P_*ylaL*_-*lacZ* activities; **E** P_*ygaO*_-*lacZ* activities; **F** P_*ykaA*_-*lacZ* activities. **G** P_*ywzA*_-*lacZ* activities; **H** P_*yaaI*_ -*lacZ* activities; **I** P_*gtaB*_ -*lacZ* activities

Eight predicted SigB PBMs (P_*ytoQ*_, P_*pucI*_, P_*ylaL*_, P_*ygaO*_, P_*ykaA*_, P_*ywzA*_*,* P_*yaaI*_, P_*gtaB*_) and the well-known P_*rsbV*_ were selected as representatives from different promoter categories (indicated in bold in Supplementary Table S2). The (putative) functions of these genes are presented in Table 1. SigB-dependent activities of translational promoter-*lacZ* fusions were determined in wt and *ΔsigB* mutants by measuring β- galactosidase activity upon temperature upshift from 37 °C to 48 °C, exposure to 4% ethanol, and exposure to 6% NaCl (in three independent experiments) (Fig. 4).

#### Category I: P_*rsbV*_ as the positive control

The *rsbV* gene is a well-known SigB regulon gene. Thus, P_*rsbV*_*,* which encompasses the sequence (GTTTAA-N_14_-GGGTAT) that exactly matches the SigB PBM consensus was used as the positive control and the Category I representative in the experimental validation. Exposure of wt cells containing P_*rsbV*_*-lacZ* to heat, ethanol, and salt resulted in 8-fold, 15-fold, and 6-fold induction of β-galactosidase activity compared to unstressed cells, respectively (Fig. 4A). In the *ΔsigB* mutant, no P_*rsbV*_ -dependent β-galactosidase activity was observed in either stressed or unstressed conditions, showing that the observed promoter activity could be attributed to SigB induction, as expected for this positive control.

#### Category II: P_*ytoQ*_ shows SigB-dependent induction under ethanol stress, P_*pucI*_ with longer spacer showed very mild SigB-dependent activity

Both *ytoQ* and *pucI* are newly identified putative SigB regulon genes in this study. P_*ytoQ*_ and P_*pucI*_*,* with predicted SigB PBM of (GTTTAA-N_14_-GGGTGA) and (GTTTAA-N_17_-GGGAAA), respectively, were used as the Category II representatives. These sequences have exact matches with the consensus − 35 binding site and both contained three conserved GGG bases at the − 10 binding site but have spacer lengths of 14 and 17 nucleotides, respectively.

Cells containing the P_*ytoQ*_*-lacZ* (GTTTAA-N_14_-GGGTGA) showed SigB-dependent l*acZ* induction only upon exposure to 4% (v/v) ethanol, but not upon temperature upshift from 37 °C to 48 °C nor upon NaCl shock of 6% (v/v) (Fig. 4B). This gene is likely also regulated by at least one other regulator because the baseline activity of P_*ytoQ*_ at T_0_ before stress exposure was already ~ 100 MU/mg protein. The average P_*ytoQ*_ -dependent β-galactosidase activity after ethanol treatment was around 230 MU/mg protein for the wt carrying P_*ytoQ*_*-lacZ*, which was about 130 MU/mg protein more than the activity at T_0_. The increase was lower (around 40 MU) in the Δ*sigB* mutant carrying P_*ytoQ*_*-lacZ*. This difference between the wt and the Δ*sigB* mutant may imply that the product of the *ytoQ* gene is specific in response to stress caused by ethanol. Although the function of the *ytoQ* gene has not been fully elucidated, it was shown to be important under vitamin B6 starvation in *B. subtilis* [59]. Using the transcription regulator database DBTBS [6], we found that the predicted SigB PBM for *ytoQ* also contained two alternative PBMs: the first was for *xre*, which is the repressor of a phage-like bacteriocin, and the second was for *codY,* the repressor involved in the response to branched-chain amino acid limitation (see Table 1). These findings indicate that *ytoQ* may be regulated by SigB, CodY, and Xre under different conditions.

A mild, yet notable SigB-dependent response was observed for *pucI* (encoding allantoin permease) upon exposure to ethanol and NaCl stresses (Fig. 4C). After cells were exposed to 6% NaCl, β- galactosidase activities of wt P_*pucI*_-*lacZ* cultures increased around 4-fold compared to the control at T_0_. However, in the Δ*sigB* P_*pucI*_-*lacZ* cultures, this increase was notably higher (~ 6-fold), suggesting that SigB may partially involve in the negative regulation of *pucI.* Upon exposure to ethanol stress, β- galactosidase activities of wt P_*pucI*_-*lacZ* cultures did not change compared to the activity at T_0_, but the Δ*sigB* P_*pucI*_-*lacZ* mutant showed ~ 2- fold higher β-galactosidase activities than the wt P_*pucI*_-*lacZ* (see Fig. 4C). This result indicated that the mild increase in β-galactosidase activities as observed for the Δ*sigB* P_*pucI*_-*lacZ* mutant did not result from the exposure to ethanol but the deletion of the *sigB* gene.

The promoter of *pucI* was induced by NaCl in the wt and further induced in the Δ*sigB* mutant, implying that *pucI* may be co-regulated by other regulators/sigma factors as found in Table 1. *pucI* may also have roles in other stress conditions, triggered by other undiscovered stressors, or stressors other than heat, ethanol, and salt. It is noteworthy that the predicted SigB PBM of P_*pucI*_ has an extended spacer (17 nucleotides) between the − 35 and − 10 binding motifs compared with the consensus spacer. This longer spacer region may affect the promoter strength by influencing the binding of the RNA polymerase, thereby affecting transcription. In transcription initiation, the bacterial RNA polymerase first locates the promoter, and its largest subunit (β-zipper) will interact with the spacer between the − 35 and − 10 elements to form a holoenzyme complex [60, 61].

#### Category III: P_*ylaL*_ showed mild SigB-dependent activity, P_*ygaO*_ and P_*ykaA*_ did not show changes and P_*ywzA*_ showed ethanol-specific SigB-dependent induction

For category III, P_*ylaL*_ (GTTTAT-N_16_-GGGAAT), P_*ygaO*_ (GTTTAT-N_14_-GGGAAT), P_*ykaA*_ (GTTTTT-N_12_-GGCTAT), and P_*ywzA*_ (GTTTAT-N_14_-AGGAAT) were selected based on the conserved GTTT at the − 35 binding site and the GG at the − 10 binding sites and different spacer lengths. *ylaL, ygaO* and *ykaA* are newly identified in this study to be potential SigB regulon candidates, in which *ywzA* is a known SigB regulon gene, used as a control of Category III.

Mild β-galactosidase activities were observed for P_*ylaL*_ but were unrelated to the stressors (Fig. 4D). In the wt P_*ylaL*_*-lacZ* culture at T_0_ (before the exposure to heat, ethanol, or salt), no β-galactosidase activity was measured, whereas the Δ*sigB* P_*ylaL*_-*lacZ* culture showed higher levels. The same results were observed in wt and the Δ*sigB* mutant after exposure to all three stressors, indicating that SigB may negatively affect *ylaL*. Based on the NCBI BlastP results, YlaL is 99.9% similar to the peptidyl-prolyl cis-trans isomerase and located next to the spore germination gene *ylaJ*. The predicted SigB PBM sequence for *ylaL* was also found to have a positive hit to the SigG binding motif with the consensus TGCATAT-N16-GATACTTA (DBTBS) (see Table 1), implying that YlaL may be coregulated by SigB and SigG, and may have a role in sporulation. The role of isomerase in sporulation was described in *B. subtilis* subs. *spizizenii* [49]*,* and the involvement of SigB in sporulation control was also reported before. SigB is known to induce the expression of *spo0E,* which is a suppressor of *spo0A* and *spoIIE* genes which are required for sporulation initiation [14, 62]. Similarly, as indicated above for P_*pucI*_, the P_*ylaL*_ activities were relatively low, which may be attributed to the long spacer length of 16 nucleotides. Further experiments are thus needed to elucidate the potential regulation of PucI and YlaL by SigB.

P_*ygaO*_ and P_*ykaA*_ did not show evident changes in transcriptional activation in the three tested conditions despite having a relatively conserved SigB PBM (Fig. 4E and F). Baseline β-galactosidase activities for both promoters were seen before stress imposition at T_0_, and the P_*ygaO*_ and P_*ykaA*_ activities in Δ*sigB* mutant did not differ from the wt upon exposure to heat, ethanol, or salt stress. Results suggested that both *ygaO* and *ykaA* are likely controlled by other regulators than SigB (Table 1). Nonetheless, as only the three most commonly used SigB stressors were tested in this study, P_*ygaO*_ and P_*ywzA*_ may respond to other environmental or nutritional stressors.

In addition, the promoter activities of *ywzA* (a known SigB regulon gene predicted with a Category III SigB PBM) were verified via experiments. The β-galactosidase activity in wt P_*ywzA*_*-lacZ* cultures increased 2-fold after heat treatment compared to cultures without treatment, but the induction after ethanol treatment stood out (20-fold higher) (Fig. 4G). No β-galactosidase activities were seen in the Δ*sigB* P_*ywzA*_*-lacZ* cultures upon heat and ethanol stress, indicating that SigB was responsible for the increased expression in the wt cultures. Exposure to osmotic stress did not lead to changes in β-galactosidase activities in wt and Δ*sigB* P_*ywzA*_*-lacZ* cultures*.* As the deletion of *sigB* abolished the activity of P_*ywzA*_ completely, *ywzA* is likely not co-regulated by other regulators under the conditions tested. However, alternative binding sites for *codY* (repressor in response to branched-chain amino acid limitation) and *araR* (repressor of the L-arabinose metabolic operon) were found for the predicted SigB PBM of *ywzA* (Table 1).

Of the newly predicted SigB regulon genes described so far (*ytoQ, pucI, ylaL, ygaO,* and *ykaA*), none of these five were identified as SigB regulated in previous studies, either via transposon mutagenesis [63–65], gel-based proteomics [30, 66–69], consensus promoter search [9], transcriptional profiling [2, 3], the combination of microarray and machine learning algorithm in defining the SigB regulon structure [5] or SigB modeling [8]. Our data show that three out of the five have SigB-dependent promoter activity, indicating that these genes may have been overlooked in earlier studies. The predicted P_*ygaO*_ and P_*ykaA*_ SigB PBM with high confidence did not show a clear SigB-dependent activation, suggesting that they might be induced by other stressors than heat, ethanol, or NaCl, or that the control by SigB is affected by the consensus and the spacer of the promoter.

#### Category IV and V: P_*yaaI*_ and P_*gtaB*_ showed SigB-dependent activities despite deviating considerably from the consensus

Lastly, P_*yaaI*_ (GTTTTT-N_14_-GGCTAC), and P_*gtaB*_ (GTTTTA-N_14_-CTTGTTTAA) were included as a representative from category IV and category V, respectively. Both *yaaI* and *gtaB* are known SigB regulon genes but were selected for verification in this study because the predicted SigB PBMs deviate considerably from the original SigB consensus (“*Plasmids and reporter strains construction*” section).

The P_*yaaI*_ *-*LacZ activity in wt was induced the most upon exposure to ethanol stress, with a 35-fold increase, and heat stress resulted in a 4-fold increase compared with untreated wt P_*yaaI*_*-lacZ* cultures, wherease no difference of wt P_*yaaI*_ *-*LacZ activity was observed under salt stress (Fig. 4H). This result suggests that the general stress gene *yaaI* plays an important role in protecting cells from damage caused by ethanol. The deletion of the *sigB* gene diminished the activity of P_*yaaI*_ under all three conditions that were tested, suggesting that the expression of *yaaI* may be solely-dependent on SigB. However, an alternative binding site for *codY* was also found for the predicted SigB PBM of *yaaI* (Table 1), but the interaction of SigB, CodY and YaaI is yet to be explored.

The β-galactosidase activities of P_*gtaB*_ were also investigated in wt and Δ*sigB* mutant (Fig. 4I). Petersohn et al. [3] reported that the putative SigB PBM for *gtaB* is located inside the gene coding region, but this study identified that the SigB PBM for *gtaB* is located upstream of the AUG start codon, containing the sequence GTTTTA-N_14_-GCTTGTTTAA. This SigB PBM met the selection criteria (“*Sigma B (SigB) promoter binding motif (PBM) reconstruction*” section) only because of the duplicated GTTT sequence at both − 35 and − 10 binding sites and it was chosen as a target to verify if SigB could bind to this predicted SigB PBM.

The wt P_*gtaB*_*-lacZ* culture displayed SigB-dependent induced β-galactosidase activity upon exposure to ethanol and salt stress despite the poor binding motif (Fig. 4I). At T_0_ before stress exposure, the baseline P_*gtaB*_ activity in wt cells was already around 290 MU/mg protein, indicating that other transcriptional regulators may co-regulate this gene. A promoter sequence search using the DBTBS database revealed binding sites for two other alternative regulators, namely, SigA and DegU (Table 1). Despite the high baseline activity, an increase in P_*gtaB*_-dependent β-galactosidase activity was seen after ethanol (584 MU) and salt shock (333 MU), but no significant increase was observed upon heat treatment. Notably, the P_*gtaB*_-*lacZ* activity in the Δ*sigB* mutant at T_0_ was also ~ 3-fold lower than in the wt, indicating that SigB might play a role in regulating *gtaB* even under the control (presumably unstressed) condition. No induction was seen in the Δ*sigB* P_*gtaB*_-*lacZ* mutant in response to ethanol, salt, and heat stress. These results are in line with available transcriptomics data for *B. subtilis* 168 wt and Δ*sigB* mutant, showing more profound expression of *gtaB* upon ethanol or salt shock than upon heat shock [3]. This example demonstrated that SigB recognizes the predicted binding sequence, at least weakly, despite the large deviation from the SigB consensus sequence (GGGTAT) at the − 10 binding site.

The identification of putative SigB regulon members (section “*Two-step SigB promoter binding motif derivation in B. subtilis 168*”), of which nine predicted SigB PBMs of Category I-V were validated in section “*Experimental validation of predicted SigB promoter binding motifs*”, suggests that the SigB regulon in *B. subtilis* 168 may be even more extensive than currently thought. The number of theoretical SigB regulon genes was recently estimated to be 411 [8], and taken together with the predicted genes in this study, the total number may exceed 500 (Fig. 3). This large number of SigB regulon genes aligned with the notion that many SigB-regulated genes are also co-regulated by other transcriptional regulators, interlinking SigB regulation with other cellular processes [8]. Category III representatives that did not show SigB dependence in this study may respond to other so far unknown stressors or their promoter activities may be affected by the spacer length and/or compositions of the promoter, which requires further confirmation.

### Functional distribution of known and predicted SigB regulon genes

**Fig. 5:**
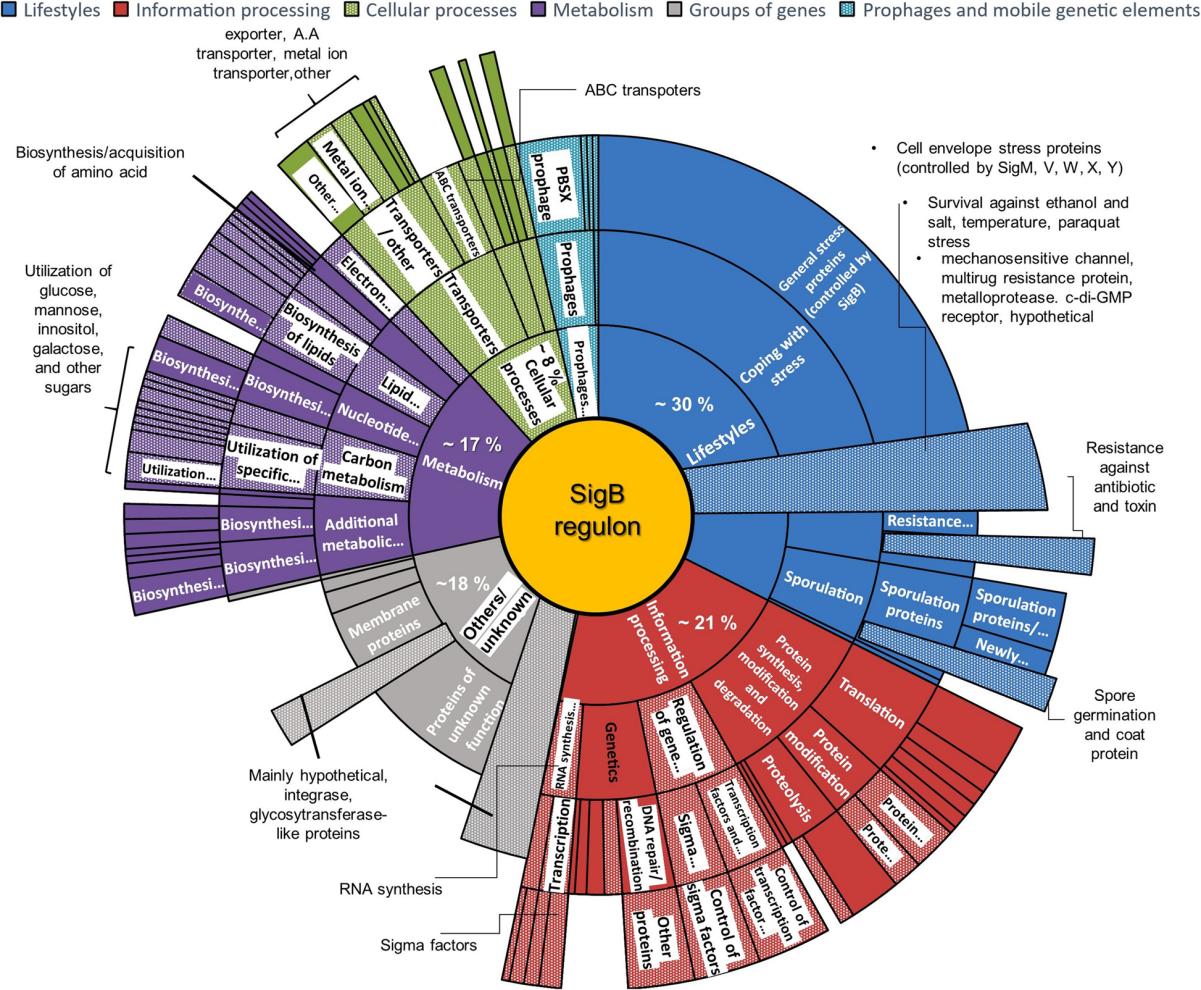
Functional distribution map for the predicted and existing SigB regulon members. The sunburst map shows five known functional groups and a group with unknown functions, each labeled with different colors, of the predicted and existing SigB regulon members. The dotted regions in the map refer to genes with either a known SigB PBM or a predicted SigB PBM in each functional category. Blue represents genes involved in lifestyles (*e.g.,* coping with stress, sporulation) (~ 30%); Red represents genes for information processing (*e.g.,* protein synthesis and modification, transcription, or translational regulation) (~ 21%); Purple indicates genes for metabolism regulation (*e.g.,* biosynthesis of amino acid, lipids, utilization of carbon sources) (~ 17%); Green indicates genes for cellular processing (*e.g.,* transporter, exporter, homeostasis) (~ 8%); Turquoise includes genes for phage-related function (~ 6%); and Grey indicates genes with unknown functions (~ 18%). Underlying background data are shown in Supplementary Table S6

The functions of the 156 predicted SigB regulon candidates in this study and all genes listed in Subtiwiki are presented in a functional distribution map (see Fig. 5). The list of genes with known functions (data extracted from Subtiwiki) is presented in Supplementary Table S2b. The sunburst map illustrates genes with and without SigB PBM (with shaded regions indicating genes with a predicted SigB PBM in this study; Fig. 4), and indicates that 30% of the genes encode for proteins involved in lifestyles (*e.g.,* coping with stress, sporulation), 21% in information processing (*e.g.,* protein synthesis and modification, transcription or translational regulation), 17% in metabolism regulation (*e.g.,* biosynthesis of amino acid, lipids, utilization of carbon sources), 8% in cellular processing (*e.g.,* transporter, exporter, homeostasis), 6% in phage-related function, and 18% constitute proteins with unknown functions.

Many of the SigB regulon members that are involved in lifestyle management have generic functions in general stress protection. Some genes are likely regulated by SigB directly as SigB PBMs were predicted; some encode proteins with a role in resistance to toxins or antibiotics and others are linked to sporulation. Among the ~ 21% of members involved in information processing, many are well-known SigB-dependent genes (such as *ctc, rsbV, rsbW,* and *sigB* itself) that are involved in the regulation of gene expression, and many play a role in protein synthesis, modification, and degradation as well as DNA repair and recombination. Interestingly, a range of genes that have a putative SigB PBM are involved in cellular processes relating to nutrient transport, such as ABC transporters, ions transporters, and amino acids transporters. Other metabolic genes that are responsible for the biosynthesis of lipid synthesis, acquisition of amino acids, and utilization of different carbon sources also have predicted SigB PBMs (Fig. 5). The remaining genes were either prophages and mobile genetic elements, or genes encoding membrane proteins with undefined functions. Further functional investigation of these genes may help to better understand their involvement in stress response regulation in *B. subtilis.*

This study identified an additional 156 SigB regulon candidates with a putative SigB PBM in *B. subtilis* based on a computational approach using a more plastic SigB PBM. Multiple factors may have limited the detection of such genes in previous studies: 1) The use of a more restricted SigB PBM in earlier studies [6, 7]; 2) Utilization of the same stressors in global SigB- mediated GSR studies. Ethanol, heat, and salt were used mostly because of their potent SigB triggering response. Although other investigators studied the induction of SigB by acid, cold, antibiotics, reduced ATP, GTP, low oxygen, glucose limitation, blue light, red light, carbonyl cyanide m-chlorophenylhydrazone (CCCP), butanol, pH, low pressure, high-level iron, and oxidative stress, global transcriptomic or proteomic analyses were not performed in all studies [3, 5, 10, 14, 30, 31, 70–80]. Therefore, SigB-regulated genes that are specific for other stressors may have not been detected. Moreover, stimuli or stressors that play a role in unexplored ecological niches may trigger SigB as well; 3) Analysis restricted to the SigB induction response. The standard setting in many studies of the global transcriptomics or proteomics SigB stress response focused on the induction pattern of SigB, and thus often checked for the “loss-of-gene-function” in the Δ*sigB* mutant. Such approaches may overlook genes that may be negatively regulated by SigB; 4) SigB is likely active without “triggers” in the control condition and co-regulates other cellular mechanisms than the SigB GSR. Several known or predicted SigB candidate genes with SigB PBMs are involved in miscellaneous functions in *B. subtilis*, for instance, biofilm formation, sporulation, utilization of sugars, biosynthesis of amino acids, and homeostasis. Reder et al. [14] and Rothstein et al. [62] reported the negative regulation of SigB in sporulation initiation. Bartolini et al., [15] demonstrated the role of SigB in regulating biofilm growth rate via the interaction with the SinR transcriptional regulator. SigB was also shown to indirectly affect the expression of surfactin, a cyclic lipopeptide (biosurfactant) [81].

Other than the functions reported in *B. subtilis,* SigB can influence motility, virulence, and invasiveness in other Bacillales members, e.g., *L. monocytogenes* and *S. aureus* [78, 82, 83], indicating that the structure of SigB regulons between species may have diverged due to differences in physiological responses upon exposure to a broad range of stressors. Thus, to obtain a global outlook of the SigB general stress regulon in other species in the Bacillales order, putative SigB regulons of 18 other *B. subtilis* strains and 106 Bacillales genomes were predicted as described in section “*Bacillales core genome phylogenetic tree reconstruction, species-specific SigB PBM, and regulon structure prediction*”.

### SigB regulon prediction for *B. subtilis* wild isolates and Bacillales genomes

**Fig. 6:**
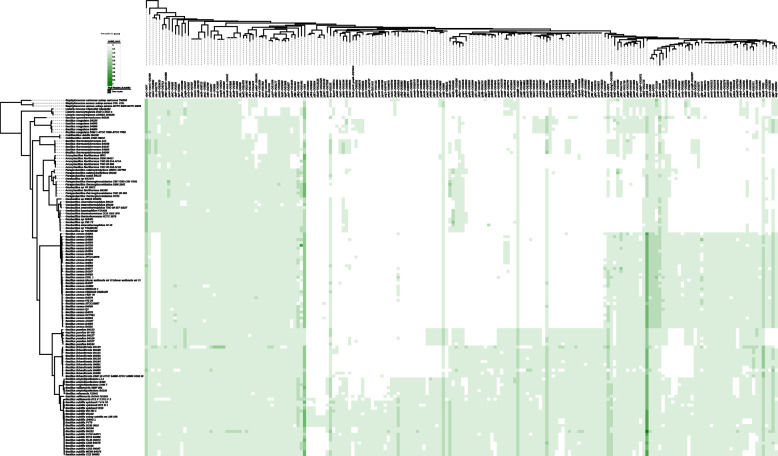
Genome tree heat map of 18 other *B. subtilis* strains and 106 Bacilalles genomes and the prediction of the presence of orthologs of *B. subtilis* 168 SigB regulon genes The phylogenetic genome tree heat map for 19 *Bacillus subtilis* genomes (including *B. subtilis* 168) and 106 Bacillales members was generated using iTOL (PMID27095192) [54]. The Y-axis in the genome tree shows the phylogenetic relationships of all genomes based on the core conserved protein sequences in each genome. The x-axis shows the clustering of *B. subtilis* 168 SigB regulon genes listed in Subtiwiki up to October 2020 (Supplementary Table S1). A green square indicates the presence of a target gene, the intensity of the green color indicates the gene copy number, and white indicates the absence of a gene. Underlying background data are shown in Supplementary Table S5

Genomes of 18 *B. subtilis* wild isolates and 106 other Bacillales genomes including different *Bacillus* species, *Listeria* spp., and *Staphylococcus* spp. (Supplementary Table S5) were mined for the presence of SigB regulon members that had been identified in *B. subtilis* 168 (Supplementary Table S1). Based on the conserved protein sequences, the reconstructed phylogenetic tree heat map in Fig. 6 showed that nearly all genes that belong to the SigB regulon in *B. subtilis* 168 had orthologs in 18 other wild *B. subtilis* isolates, except for a small cluster of germination genes (*yfkR, yfkS, yfkT*) and a group of genes with unknown function (*ykzN, ypuB, yydC*) (details in Supplementary Table S5). Much more prominent differences were seen between SigB regulons of *B. subtilis* and other *Bacillus* species and Bacillales genomes, such as *B. licheniformis, B. cereus,* and other further related species like *Geobacillus, Listeria,* and *Staphylococcus.* Around 25% of the *B. subtilis* 168 SigB regulon genes were absent in *B. licheniformis,* around 50% were missing in *B. cereus,* and three quarters were lacking in *Geobacillus, Listeria,* and *Staphylococcus* (Fig. 6, Supplementary Table S5).

The prediction results showed that SigB regulates different sets of genes in different species, *e.g.,* in *B. cereus, L. monocytogenes,* or *S. aureus.* Therefore, the SigB PBMs are likely species-specific and deviate from the SigB consensus of *B. subtilis* (GTTTAA-N_15 (± 2 bp)_ -GGGTAT), or have the same PBM but is/are not present in front of the same genes. This assumption was further investigated by reconstructing species-specific SigB PBMs per inspected species (described in section “*Species-specific SigB PBMs and SigB regulon structures prediction for other Bacillales genomes*”) which is illustrated in Fig. 2. In a step-wise approach, 1) orthologous genes of the *B. subtilis* 168 SigB regulon members in other analyzed species were first predicted, 2) the predicted orthologous genes were grouped into operons, 3) promoters of these operons were used to reconstruct a species-specific SigB PBM, 4) the respective genome of a species was screened for the presence of the constructed species-specific PBM, resulting in a new list of genes with putative SigB PBMs, 5) operons for these genes with putative SigB PBMs were again predicted, and species-specific SigB PBM 2 was constructed, and lastly, 6) the respective genome of a species was repeatedly screened for the presence of the species-specific SigB PBM 2 with different spacers N_12_ – N_17_ (Fig. 2). The same procedures were performed for other species included in this study, and each species-specific SigB PBM was used to derive a Bacillales consensus (KTTTW- N_12_-N_17_- GGGWAW). The Bacillales consensus is less conserved at the first guanine nucleotide in the − 35 region when compared with the *B. subtilis* SigB consensus and contains more thymine than adenine nucleotides.

The predicted species-specific SigB regulon members with/without SigB PBM are presented in a heat map (Supplementary Fig. S1), showing the 1) absence/presence of predicted genes that are orthologous to the *B. subtilis* 168 SigB regulon members in 124 Bacillales genomes, and 2) the putative species-specific SigB regulon genes with or without a predicted SigB PBM. The full list of these genes is shown in Supplementary Table S6. Four major observations can be made based on the heat map: 1) many other Bacillales genomes contain genes that are orthologous to the SigB regulon members of *B. subtilis* 168, but they do not necessarily have a SigB PBM; 2) groups of predicted regulon genes with/without SigB PBM are species-specific; 3) a group of genes that are orthologous to the SigB regulon members of *B. subtilis* 168 or the predicted species-specific regulon genes do not have a SigB PBM, and lastly 4) a group of genes with/without SigB PBM are specific for *Bacillus* species but is absent in other Bacillales genomes. These results suggested two different possibilities for different species: divergent SigB PBMs or conserved PBMs may control different genes.

Overall, the results obtained confirm that SigB plays a role in adaptive stress response in many species, but that the actual cellular responses and genes involved are different for different species. Species-specific SigB regulons may correspond with distinct physiological responses of species when dealing with a broad range of stressors in their environments. Orthologs of SigB-regulated genes with a SigB PBM as found in *B. subtilis* were mainly found in other *Bacillus* species but did not necessarily contain an upstream SigB PBM, and the majority of the *B. subtilis* SigB regulon genes were absent in *Listeria* spp. and *Staphylococcus* spp.

These results are in line with the studies of Scott et al. [16], who reported on the divergence of the SigB GSR regulons within the *B. cereus* sensu *lato* group (containing species that are not included in this study: *B. anthracis, B. mycoides, B. pseudomycoides, B. thuringiensis, B. weihenstephanensis and B. cytotoxicus*). Four lineages of the SigB regulon were described in their study, and each lineage has arisen from the selection of a set of genes from the common gene pool, with the “reassignment” of a SigB promoter to these genes to support pathogenesis for different sensu *lato* members [16]. The extra members in addition to the SigB core regulon (consisting of ~ 20 members) was suggested to serve a distinct function in different habitats and support the phenotype of a specific member, such as enhancing pathogenic potential or increasing competence against other microorganisms in the soil [16]. Moreover, the SigB PBM predicted for *B. cereus* in this study (shown in Fig. 2) is highly similar to the one described in the study of Scott et al. [16] despite using different species of *B. cereus* group members (in this study only *B. cereus* genomes were used, Supplementary Table S6).

### The Occurrence of SigB sensing modules in other *B. subtilis* strains and Bacillales genomes

Our analysis showed differences between the predicted SigB regulons for various Bacillales genomes. We furthermore examined the presence of the three well-known SigB signaling modules in Bacillales, i.e. RsbRST, RsbQP, and RsbKY (as described in section “*Bacillales Sigma B (SigB) sensing modules prediction*”).

**Fig. 7:**
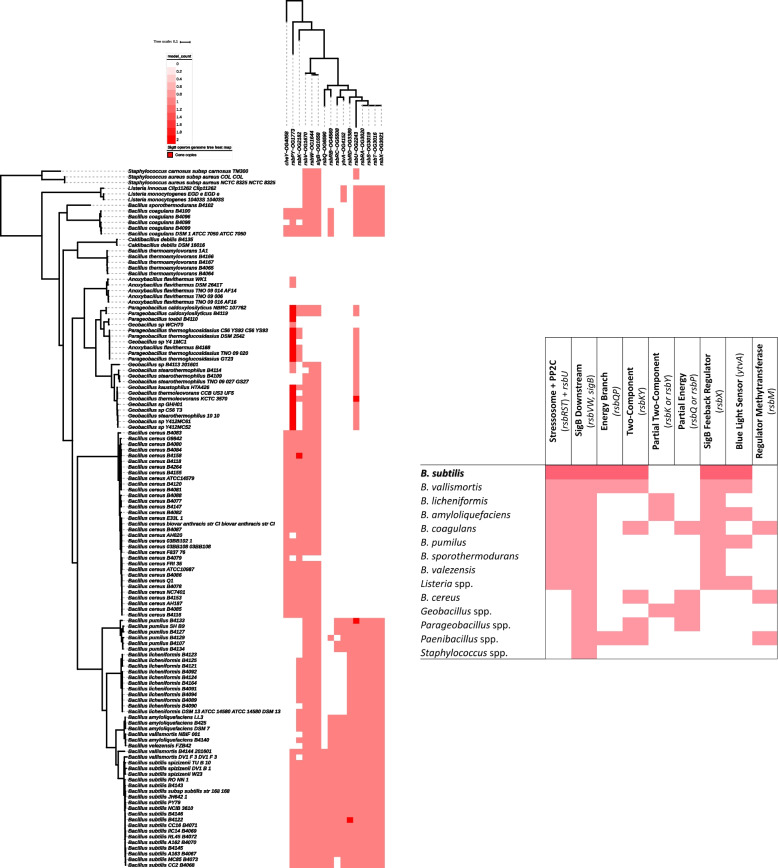
Genome tree heat map of sensing modules of the SigB general stress for 19 *Bacillus subtilis* genomes and 106 Bacillales members. The heat map of the core genome tree of sensing modules of the SigB general stress for 19 *Bacillus subtilis* genomes and 106 Bacillales members was generated using iTOL (PMID27095192) [54]. The tree on the left shows the phylogenetic relationships of all genomes based on the core conserved protein sequences in each genome. The tree on the top shows the clustering of genes involved in SigB signal sensing. The red squares indicates the presence of a target gene, and the intensity of the red color indicates the gene copy number. White indicates the absence of a gene. Underlying background data are shown in Supplementary Table S7. The insert shows the summary of the general distribution of the three sensing modules for each species belonging to the Bacillales order. Stressosome refers to the *rsbRST* stressosome genes and stressosome downstream elements to *rsbV, rsbW* and *sigB*. The energy branch refers to the *rsbQP* genes and the two-component to the *rsbKY* genes. Inspected strains that contain only a single gene of a signaling module, *e.g.,* the presence of an orphan *rsbK* gene without its cognate response regulator gene *rsbY,* or the presence of a *rsbP* phosphatase gene without its partner *rsbQ*, are referred to as having a partial two-component, or a partial energy system, respectively. Other SigB sensors and regulators included the blue light sensor (YtvA), the regulator of SigB methyltransferase BC1007 (renamed to RsbM by Chen et al., [27]), and the feedback regulator RsbX phosphoserine phosphatase. Underlying background data are shown in Supplementary Table S7

The absence/presence of genes involved in sensing stressors, SigB transduction, activation, and regulation in 19 *B. subtilis* genomes and 106 Bacillales members is presented in Fig. 7. The complete datasets relating to the presence of the sensing modules are presented in Supplementary Table S7. The majority of the inspected genomes carried the *sigB* gene, however, it was absent in several species like *B. thermoamylovorans, Parageobacillus thermoglucosidasius,* and *Anoxybacillus.* These species likely evolved to utilize other stress sensing systems and were therefore excluded from further analyses.

All species that were found to contain the *sigB* gene also carried the *rsbV* and *rsbW* genes. These three genes are highly conserved in Bacillales, including the *Geobacillus* and *Paenibacillus* species that have a high GC content. In general, the complete stressosome system (*rsbRST* and *rsbU*) and its feedback regulator (*rsbX*) were found in *B. subtilis, B. vallismortis, B. licheniformis, B. amyloliquefaciens, B. coagulans, B. pumilus, B. valezensis, B. sporothermodurans, L. monocytogenes,* and *L. innocua* (Fig. 7). The RsbQP module was found in *B. subtilis, B. vallismortis,* and *Paenibacillus* spp. and the two-component system (*rsbKY*) and its regulator *rsbM* were identified in *B. cereus, Paenibacillus* spp. and *B. coagulans* (Fig. 7)*.*

#### The stressosome RsbRST SigB activation pathway

The RsbRST stressosome system was detected in many species but only the RsbRA ortholog is conserved (Fig. 7). Many species that contain the stressosome genes lacked one or more genes that encodes either the RsbRB, RsbRC, RsbRD or the YtvA ortholog. *B. licheniformis, B. pumilus,* and *B. sporothermodurans* did not contain the genes that encode the RsbRB and RsbRC orthologs, whereas *B. coagulans* did not have the genes encoding the RsbRC and RsbRD orthologs. Only *B. subtilis, B. vallismortis, B. amyloliquafaciens, B. pumilus, L. monocytogenes,* and *L. innocua* contained the gene encoding the fifth RsbR ortholog, YtvA, which is involved in the sensing of blue light. Even in *B. subtilis*, not every strain carried the genes encoding the same RsbR orthologs. Some of the *B. subtilis* food isolate strains (B4068 and B4073) lost the gene encoding the RsbRC ortholog and *B. subtilis* B4122 had two gene copies encoding the RsbRD ortholog (Fig. 7, Supplementary Table S7).

The occurrence of different types and numbers of RsbR orthologs results in the formation of heterogeneous stressosome complexes [84], thereby affecting specific stress sensing via the turrets (referring to the protein structure of the RsbR and its paralogs) with different ligands [17, 20]. Moreover, from an evolutionary point of view, different species may be exposed to certain stress conditions in particular ecological niches, putting selective pressures on retaining certain RsbR orthologs that are needed for sensing of a specific stress. Different RsbR orthologs can have distinct functions in mediating stress; RsbRC has for instance been shown to be responsible for a slow progressive stress response upon ethanol stress, whereas RsbRA mediates a fast transient response [85]. However, RsbRC was absent in most genomes that carry genes which encode stressosome members (Fig. 7), which implies that its role may not be essential, or that its function is redundant in the presence of RsbRA. In *B. subtilis*, it was shown that RsbRA and RsbRB orthologs are responsible for light sensing, and the role of RsbR can be complemented by RsbRC or RsbRD [86].

Additionally, the gene *rsbX* was found only in strains that contained the stressosome genes, confirming the reported function of the RsbX in forming a negative feedback loop by dephosphorylating the RsbR and RsbS, thereby resetting the activated stressosome to its original state [87]. The absence of *rsbX* in other strains that do not contain the stressosome genes was not a surprise as this feedback loop was probably not required.

#### The bipartite RsbQP SigB activation pathway

The entire RsbQP module was detected in *B. subtilis, B. vallismortis,* and *Paenibacillus* spp. (Fig. 7), indicating that the nutritional stress sensing branch is not restricted to just *B. subtilis*. The *rsbQ* gene was missing in the majority of the genomes, but the *rsbP* gene, encoding a PP2C phosphatase, was distributed more broadly. As *rsbP* and *rsbY* belong to the same OG1773 group, a “*rsbP*” ortholog was also found in the genomes of *B. cereus, Geobacillus* spp., *Paenibacillus* spp., and *Parageobacillus* spp. (see Fig. 7).

The finding was in line with the publication of Nadezhdin et al. [25] who suggested that the predicted RsbQP could be functional in other species, or react with other stressors, but not in the same way as described for *B. subtilis* [75, 77]. The observation that RsbQ is generally absent while RsbP is generally present in Bacillales (Supplementary Table S7) suggests RsbP may have other so far unidentified functions, and its interaction partner may not be limited to RsbQ. This speculation can be supported by reports on alternative functions of RsbP in earlier studies, which showed that RsbP is also involved in sensing red light [77] and that this protein interacted with the stressosome to mediate resilience toward oxidative and nitrosative stress in *B. subtilis* [88].

#### The two-component RsbKY SigB activation pathway

The two-component system encoded by *rsbKY* and *rsbM* was found in *B. cereus, Paenibacillus* spp., and *B. coagulans*. This sensing module was well-known to be specific to *B. cereus* and its group members [57], while a full RsbKYM system in *B. coagulans* has not been described previously. In addition, the *rsbK* (*bc1008*) gene in *B. cereus* was found to have an ortholog in *B. subtilis* and other group members like *B. licheniformis, B. amyloliquefaciens, B. pumilus,* and *B. vallismortis* (Fig. 7, Supplementary Table S7), but no cognate response regulator was detected adjacent to the predicted *rsbK* gene in these species. The functionality of an RsbK ortholog in *B. subtilis* and its potential role in the SigB activation pathway remains to be confirmed.

The suggestion of an alternative SigB activation pathway in *B. subtilis* is not new, as extreme heat and chill conditions have been reported to induce SigB either directly, or independently from RsbV [30, 74], and nitrosative stress has been reported to trigger SigB in the absence of RsbT or RsbP [31]. Moreover, the RsbW (the anti-sigma factor) exhibited high cross-phosphorylation activity by other kinases [89], and may thereby cause unexpected SigB activation in *B. subtilis*.

## Conclusion

This study generated a SigB PBM that took spacer composition into account and has higher plasticity than the previously reported consensus sequence [6–8]. This was used to identify potential novel candidates that belong to the SigB regulon of *B. subtilis*. Of the 255 genes with predicted SigB PBMs as identified in this study, 99 genes have previously been reported in the literature, indicating the identification of 156 new putative SigB regulon members. The functionality of nine predicted SigB PBMs (including a positive control P_*rsbV*_) was further validated via experiments and results that were obtained showed that 1) some promoters containing the predicted SigB PBMs are stressor-specific; 2) spacer length likely influences the promoter activity with a spacer length of 14 bp appearing to be optimal; 3) less conserved SigB PBMs are still recognized by SigB (e.g., P_*gtaB*_) but may be co-regulated by other transcriptional regulators.

Furthermore, this study demonstrated the diversity of SigB regulons in different species of the Bacillales order. The various SigB regulons are likely linked with distinct strategies that are required for survival of different species in diverse ecological niches. While all Bacillales members can be present in soils, some inhabit salt lakes, hot springs, guts of invertebrates, etc. Thus, the strategies used to cope with stressors found in the soil environments likely overlap between these species, but different stress management strategies may be required in other niches. A Bacillales SigB consensus was predicted, with the sequence of KTT at the − 35 and the GG at the − 10 binding site, respectively. The SigB stress sensing modules were also species-specific and may even vary between different strains of the same species, likely due to the evolution of Bacillales members in specific habitats, demanding different needs to sense unique stressors.

Overall, the entire SigB regulatory network is sophisticated and not yet fully understood even for the well-characterized organism *B. subtilis* 168. Knowledge and information gained in this study can be used in further SigB GSR studies to uncover a complete picture of the role of SigB in *B. subtilis* and other species.

## Supplementary Information

**Additional file 1: Supplementary Table S1.** List of SigB regulon genes described to date October 2020 for ***Bacillus subtilis*** 168. **Supplementary Table S2.** The list of newly predicted SigB regulon genes with a SigB PBM in this study. **Supplementary Table S2b.** Functional distribution of SigB regulon candidate. **Supplementary Table S3.** Strains and plasmids constructed in this study. **Supplementary Table S4.** Oligonucleotides used in this study. **Supplementary Table S5.** Presence and absence of ***B. subtilis*** 168 SigB regulon genes described to date October 2020 in other Bacillales. **Supplementary Table S6.** Predicted putative species-specific SigB regulon genes in 19 *Bacillus subtilis* genomes and 96 Bacillales members. **Supplementary Table S7.** Presence and absence of genes involved in SigB sensing modules in Bacillales described to date October 2020.

**Additional file 2: Supplementary Fig. S1.** Heat map of SigB regulon members with SigB promoter binding motifs in 125 Bacillales.

## Data Availability

All data generated or analysed during this study are included in this published article (and its supplementary information files). The whole genome sequences of the 125 genomes used are published on the NCBI Assembly Database at https://www.ncbi.nlm.nih.gov/assembly/, with the respective genome accession numbers as shown in the list below. **Table:** 

**Genome**	**Genome accession no.**
*Anoxybacillus_flavithermus_B4168*	GCF_001587555.1
*Anoxybacillus_flavithermus_DSM_2641T*	GCF_002243705.1
*Anoxybacillus_flavithermus_TNO_09_006*	GCF_000327465.1
*Anoxybacillus_flavithermus_TNO_09_014_AF14_*	GCF_001651525.1
*Anoxybacillus_flavithermus_TNO_09_016_AF16_*	GCF_001651545.1
*Anoxybacillus_flavithermus_WK1*	GCF_000019045.1
*Bacillus_amyloliquefaciens_B4140*	GCF_001587325.1
*Bacillus_amyloliquefaciens_B425*	GCF_001587435.1
*Bacillus_amyloliquefaciens_DSM_7*	GCF_000204275.1
*Bacillus_amyloliquefaciens_LL3*	GCF_000196735.1
*Bacillus_cereus_03BB102_1_*	GCF_000022505.1
*Bacillus_cereus_03BB108*	GCF_000832865.1
*Bacillus_cereus_AH187*	GCF_000021225.1
*Bacillus_cereus_AH820*	GCF_000021785.1
**Genome**	**Genome accession no.**
*Bacillus_cereus_ATCC14579*	GCF_000007825.1
*Bacillus_cereus_B4077*	GCF_001008565.1
*Bacillus_cereus_B4078*	GCF_001008575.1
*Bacillus_cereus_B4079*	GCF_001604665.1
*Bacillus_cereus_B4080*	GCF_001008595.1
*Bacillus_cereus_B4081*	GCF_001619285.1
*Bacillus_cereus_B4082*	GCF_001619425.1
*Bacillus_cereus_B4083*	GCF_001619335.1
*Bacillus_cereus_B4084*	GCF_001619445.1
*Bacillus_cereus_B4085*	GCF_001619465.1
*Bacillus_cereus_B4086*	GCF_001008585.1
*Bacillus_cereus_B4087*	GCF_001008645.1
*Bacillus_cereus_B4088*	GCF_001619355.1
*Bacillus_cereus_B4116*	GCF_001619385.1
*Bacillus_cereus_B4118*	GCF_001619525.1
*Bacillus_cereus_B4120*	GCF_001619395.1
*Bacillus_cereus_B4147*	GCF_001008655.1
*Bacillus_cereus_B4153*	GCF_001008695.1
*Bacillus_cereus_B4155*	GCF_001619405.1
*Bacillus_cereus_B4158*	GCF_001008665.1
*Bacillus_cereus_B4264*	GCF_000021205.1
*Bacillus_cereus_biovar_anthracis_str_CI_biovar_anthracis_str_CI*	GCF_000143605.1
*Bacillus_cereus_E33L_1_*	GCF_000833045.1
*Bacillus_cereus_F837_76*	GCF_000239195.1
*Bacillus_cereus_FRI_35*	GCF_000292415.1
*Bacillus_cereus_G9842*	GCF_000021305.1
*Bacillus_cereus_NC7401*	GCF_000283675.1
*Bacillus_cereus_Q1*	GCF_000013065.1
*Bacillus_coagulans_B4096*	GCF_001587275.1
*Bacillus_coagulans_B4098*	GCF_001587225.1
*Bacillus_coagulans_B4099*	GCF_001587215.1
*Bacillus_coagulans_B4100*	GCF_001587205.1
*Bacillus_coagulans_DSM_1__ATCC_7050_ATCC_7050*	GCF_000832905.1
*Bacillus_licheniformis_B4089*	GCF_001925025.1
*Bacillus_licheniformis_B4090*	GCF_001587285.1
*Bacillus_licheniformis_B4091*	GCF_001587315.1
*Bacillus_licheniformis_B4092*	GCF_001587195.1
*Bacillus_licheniformis_B4094*	GCF_001925115.1
*Bacillus_licheniformis_B4121*	GCF_001925045.1
*Bacillus_licheniformis_B4123*	GCF_001925035.1
*Bacillus_licheniformis_B4124*	GCF_001925055.1
*Bacillus_licheniformis_B4125*	GCF_001925105.1
*Bacillus_licheniformis_B4164*	GCF_001587355.1
*Bacillus_licheniformis_ATCC_14580_DSM_13*	GCF_000011645.1
**Genome**	**Genome accession no.**
*Bacillus_pumilus_B4127*	GCF_000828345.1
*Bacillus_pumilus_B4129*	GCF_000828375.1
*Bacillus_pumilus_B4133*	GCF_000828455.1
*Bacillus_pumilus_B4134*	GCF_000828425.1
*Bacillus_pumilus_SH_B9*	GCF_001578205.1
*Bacillus_sporothermodurans_B4102*	GCF_001587375.1
*Bacillus_subtilis_A162_B4070*	GCF_000830675.1
*Bacillus_subtilis_A163_B4067*	GCF_000828495.1
*Bacillus_subtilis_B4122*	GCF_001619555.1
*Bacillus_subtilis_B4143*	GCF_000832195.1
*Bacillus_subtilis_B4145*	GCF_000830735.1
*Bacillus_subtilis_B4146*	GCF_000830645.1
*Bacillus_subtilis_CC16_B4071*	GCF_000830695.1
*Bacillus_subtilis_CC2_B4068*	GCF_000830635.1
*Bacillus_subtilis_IIC14_B4069*	GCF_000830605.1
*Bacillus_subtilis_JH642_1_*	GCF_000699465.1
*Bacillus_subtilis_MC85_B4073*	GCF_000699465.1
*Bacillus_subtilis_NCIB_3610*	GCF_000186085.1
*Bacillus_subtilis_PY79*	GCF_000497485.1
*Bacillus_subtilis_RL45_B4072*	GCF_000830595.1
*Bacillus_subtilis_RO_NN_1*	GCF_000227485.1
*Bacillus_subtilis_spizizenii_DV1_B_1*	GCF_000245035.1
*Bacillus_subtilis_spizizenii_TU_B_10*	GCF_000227465.1
*Bacillus_subtilis_spizizenii_W23*	GCF_000146565.1
*Bacillus_subtilis_subsp_subtilis_str_168_168*	GCF_000009045.1
*Bacillus_thermoamylovorans_1A1*	GCF_000751775.1
*Bacillus_thermoamylovorans_B4064*	GCF_000832245.1
*Bacillus_thermoamylovorans_B4065*	GCF_000832165.1
*Bacillus_thermoamylovorans_B4166*	GCF_000832175.1
*Bacillus_thermoamylovorans_B4167*	GCF_000832185.1
*Bacillus_vallismortis_B4144_201601*	GCF_001587405.1
*Bacillus_vallismortis_DV1_F_3_DV1_F_3*	GCF_000245315.1
*Bacillus_vallismortis_NBIF_001*	GCF_002113805.1
*Bacillus_velezensis_FZB42*	GCF_000015785.1
*Caldibacillus_debilis_B4135*	GCF_001587535.1
*Caldibacillus_debilis_DSM_16016*	GCF_000383875.1
*Geobacillus_kaustophilus_HTA426*	GCF_000009785.1
*Geobacillus_sp__Y412MC52*	GCF_000024705.1
*Geobacillus_sp_B4113_201601*	GCF_001587475.1
*Geobacillus_sp_C56_T3*	GCF_000092445.1
*Geobacillus_sp_GHH01*	GCF_000336445.1
*Geobacillus_sp_WCH70*	GCF_000023385.1
*Geobacillus_sp_Y4_1MC1*	GCF_000166075.1
*Geobacillus_sp_Y412MC61*	GCF_000024705.1
**Genome**	**Genome accession no.**
*Geobacillus_stearothermophilus_B4109*	GCF_001587495.1
*Geobacillus_stearothermophilus_B4114*	GCF_001587395.1
*Geobacillus_stearothermophilus_TNO_09_027_GS27_*	GCF_001651555.1
*Geobacillus_thermoleovorans_CCB_US3_UF5*	GCF_000236605.1
*Geobacillus_thermoleovorans_KCTC_3570*	GCF_001610955.1
*Listeria_innocua_Clip11262_Clip11262*	GCF_000195795.1
*Listeria_monocytogenes_10403S_10403S*	GCF_000168695.2
*Listeria_monocytogenes_EGD_e_EGD_e*	GCF_000196035.1
*Paenibacillus_sp__JDR_2*	GCF_000023585.1
*Paenibacillus_sp_Y412MC10_Y412MC10*	GCF_000024685.1
*Parageobacillus_caldoxylosilyticus_B4119*	GCF_001587505.1
*Parageobacillus_caldoxylosilyticus_NBRC_107762*	GCF_000632715.1
*Parageobacillus_thermoglucosidasius_C56_YS93_C56_YS93*	GCF_000178395.2
*Parageobacillus_thermoglucosidasius_DSM_2542*	GCF_001295365.1
*Parageobacillus_thermoglucosidasius_GT23*	GCF_001651535.1
*Parageobacillus_thermoglucosidasius_TNO_09_020*	GCF_000258725.1
*Parageobacillus_toebii_B4110*	GCF_001598935.1
*Staphylococcus_aureus_subsp_aureus_COL_COL*	GCF_000012045.1
*Staphylococcus_aureus_subsp_aureus_NCTC_8325_NCTC_8325*	GCF_000013425.1
*Staphylococcus_aureus_subsp_aureus_ATCC1228_ATCC1228*	GCF_000007645.1

## Abbreviations

GSR: General stress response
GOI: Gene of interest
PBM: Promoter binding motif
KO: Knockout
ONPG: Ortho-nitrophenyl-β-galactoside
