# Supplementary figures and images for: Prediction and validation of novel SigB regulon members in Bacillus subtilis and regulon structure comparison to Bacillales members
Source: BMC Microbiol. 2023 Jan 18;23:17. doi: 10.1186/s12866-022-02700-0 (PMC9847131; doi:10.1186/s12866-022-02700-0)

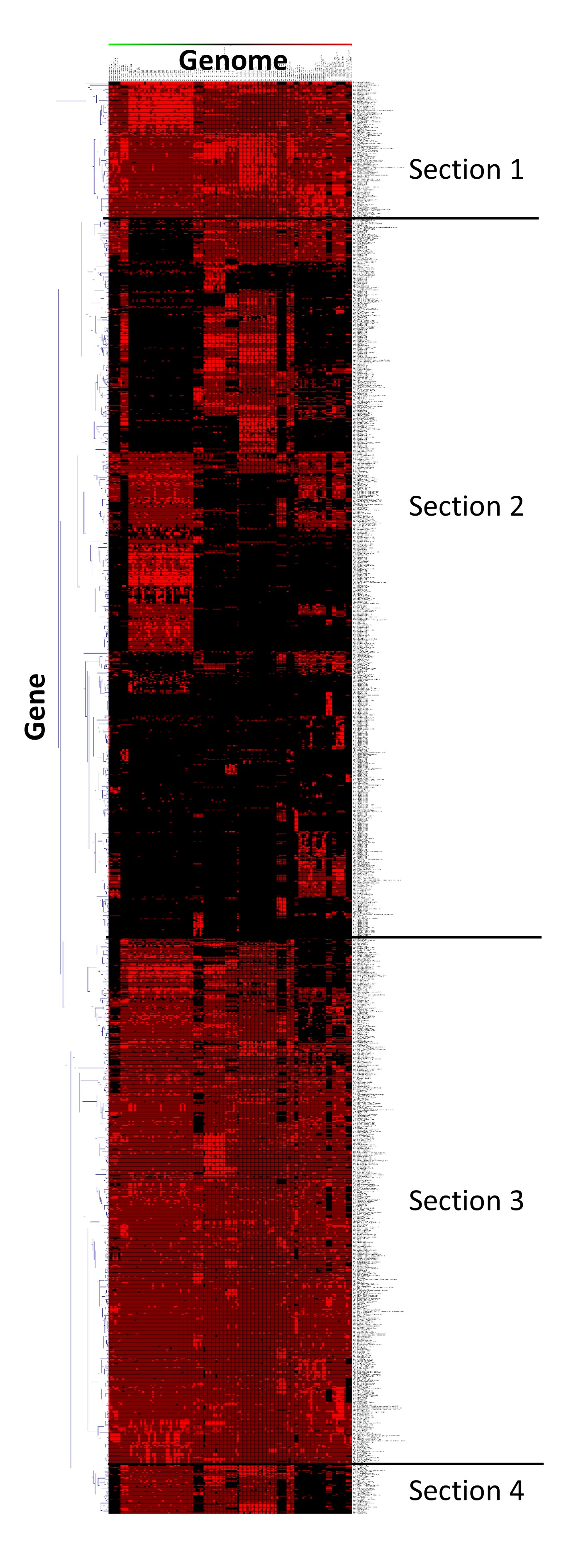

Supplement: Supplementary file 2 — Additional file 2: Supplementary Fig. S1. Heat map of SigB regulon members with SigB promoter binding motifs in 125 Bacillales. [file 12866_2022_2700_MOESM2_ESM.jpg]
